# Whole metagenome sequencing reveals links between mosquito microbiota and insecticide resistance in malaria vectors

**DOI:** 10.1038/s41598-018-20367-4

**Published:** 2018-02-01

**Authors:** Nsa Dada, Mili Sheth, Kelly Liebman, Jesus Pinto, Audrey Lenhart

**Affiliations:** 1 Entomology Branch, Division of Parasitic Diseases and Malaria, Center for Global Health, United States Centers for Disease Control and Prevention, 1600 Clifton RD. NE. MS G-49, Atlanta, GA 30329 United States of America; 2 American Society for Microbiology, 1752 N Street, N. W. Washington, D. C., 20036 United States of America; 3 Biotechnology Core Facility Branch, Division of Scientific Resources, National Center for Emerging & Zoonotic Infectious Diseases, United States Centers for Disease Control and Prevention, 1600 Clifton RD. NE, Atlanta, GA 30329 United States of America; 4 Instituto Nacional de Salud, Avenida Defensores del Morro (Ex-Huaylas) 2268, Chorrillos, Lima, Peru; 5 Present Address: Vector-Borne Disease Section, Division of Communicable Disease Control, Center for Infectious Diseases, California Department of Public Health, 850 Marina Bay Parkway, Richmond, CA 94804 United States of America

## Abstract

In light of the declining global malaria burden attained largely due to insecticides, a deeper understanding of the factors driving insecticide resistance is needed to mitigate its growing threat to malaria vector control programs. Following evidence of microbiota-mediated insecticide resistance in agricultural pests, we undertook a comparative study of the microbiota in mosquitoes of differing insecticide resistance status. The microbiota of wild-caught *Anopheles albimanus*, an important Latin American malaria vector, that were resistant (FEN_Res) or susceptible (FEN_Sus) to the organophosphate (OP) insecticide fenitrothion were characterized and compared using whole metagenome sequencing. Results showed differing composition of the microbiota and its functions between FEN_Res and FEN_Sus, with significant enrichment of OP-degrading bacteria and enzymes in FEN_Res compared to FEN_Sus. Lower bacterial diversity was observed in FEN_Res compared to FEN_Sus, suggesting the enrichment of bacterial taxa with a competitive advantage in response to insecticide selection pressure. We report and characterize for the first time whole metagenomes of *An. albimanus*, revealing associations between the microbiota and phenotypic resistance to the insecticide fenitrothion. This study lays the groundwork for further investigation of the role of the mosquito microbiota in insecticide resistance.

## Introduction

Insecticide resistance is a rapidly emerging threat to global malaria control efforts^1,2^, particularly in sub-Saharan Africa, where the greatest burden of disease lies^3^. In Latin America, regional successes in malaria control have led to a shift in focus from malaria control to malaria elimination. Vector control remains the cornerstone of malaria control and elimination programs, with a greater emphasis now being placed on understanding the role that insecticide resistance may play in compromising these elimination efforts^4,5^. Malaria vector control relies primarily on the use of indoor residual spraying (IRS) and long lasting insecticidal nets (LLINs) to reduce vector populations and protect people from potentially infectious mosquito bites^1,6^. These approaches both utilize chemical insecticides that can select for insecticide resistance in the vector populations they target^7,8^. Thus, the mechanisms leading to the evolution of insecticide resistance in malaria vectors are under extensive study, particularly given the limited number of insecticides approved for public health use^9^. To date, four key mechanisms of insecticide resistance have been described in malaria vectors: (i) target site insensitivity, where changes to the insecticide target molecules render them unsuitable for binding^9^; (ii) metabolic resistance, where heightened enzymatic activity leads to increased levels of insecticide detoxification^9^; (iii) behavioral changes resulting in the evasion of contact with insecticide treated surfaces^10,11^; and (iv) cuticle modification, which prevents or reduces the cuticular penetration of insecticides^12^.

*Anopheles albimanus*, the main coastal malaria vector in Latin America, has been reported to show resistance to multiple classes of insecticides, including organophosphates (OPs)^7,13,14^, one of the recommended classes of insecticides for IRS^6^. Two mechanisms have so far been documented for OP resistance in *An*. *albimanus*: a metabolic resistance mechanism involving elevated levels of nonspecific esterases, and a target site resistance mechanism involving acetylcholinesterase (AChE) insensitivity^14–18^. However, the mode of selection and underlying factors driving resistance to OPs and other classes of insecticides in *An. albimanus* remain largely unexplored.

Microbes colonize a wide variety of environments including the tissues of insects, where they undertake various metabolic functions, including the degradation of pesticides^19,20^. Microbes have been linked to insecticide resistance in agricultural pests^21,22^, but their role in conferring resistance in mosquitoes is unknown. The advent of new and affordable molecular tools has led to a plethora of microbiome studies, including studies of mosquito microbiota. Many of these studies have focused on characterizing the microbiota of medically important mosquito genera^23–28^, as well as identifying their role in mosquito behavior, biology, and pathogen transmission^29,30^, with a few studies suggesting their potential role in insecticide resistance^31–34^. As with most studies on insecticide resistance in malaria vectors^9^, studies of the microbiota of malaria vectors have mainly focused on sub- Saharan African and Southeast Asian mosquitoes^35^, with few such studies on Latin American vectors, particularly *An. albimanus*^36,37^. The studies of *An. albimanus* microbiota have largely focused on the effect of the microbiota on malaria parasite development, and have identified a limited number of bacterial species using culture dependent methods.

Based on the documented links between insecticide resistance and the presence of pesticide-degrading (particularly, OP-degrading) bacteria in agricultural pests^21,22^, as well as the identification of OP resistance in *An. albimanus*^17^, we hypothesized that bacteria could be contributing to insecticide resistance through increased degradation of insecticides in resistant mosquito populations. To test this, our objective was to characterize and compare the microbiota of *An. albimanus* with differing fenitrothion resistance phenotypes^14^. Specifically, bacterial compositions between fenitrothion resistant (FEN_Res) and susceptible (FEN_Sus) *An. albimanus* were compared using whole metagenome sequencing (WMS), a next generation sequencing (NGS) technique that captures both cultivable and non-cultivable bacteria as well as their putative functions. The findings presented here show differences between the microbiota of FEN_Res and FEN_Sus, as well as associations between the mosquito microbiota and xenobiotic degradation.

## Results

### Descriptive statistics

Female *An*. *albimanus* with differing fenitrothion resistance profiles were pooled (FEN_Res, 30/pool; FEN_Sus, 10/pool) and underwent WMS on the Illumina® HiSeq2500 platform. A total of 83,947,332 (FEN_Res) and 60,444,900 (FEN_Sus) raw sequencing reads were generated, with 91% and 84% of these reads passing quality control, respectively. In both samples, read lengths ranged from 60–232 bp after quality trimming, with 63% (FEN_Res) and 68% (FEN_Sus) of total reads aligning to bacterial proteins after host (*An. albimanus*) genome removal. Out of the reads that aligned to bacterial proteins, 0.9% and 0.8% aligned to bacterial xenobiotic degradation pathways in FEN_Res and FEN_Sus, respectively.

### Differential bacterial composition between fenitrothion resistant and susceptible *An. albimanus*, with significant enrichment of *Klebsiella pneumoniae*, an OP-degrading bacterial species, in resistant *An. albimanus*

**Figure 1:**
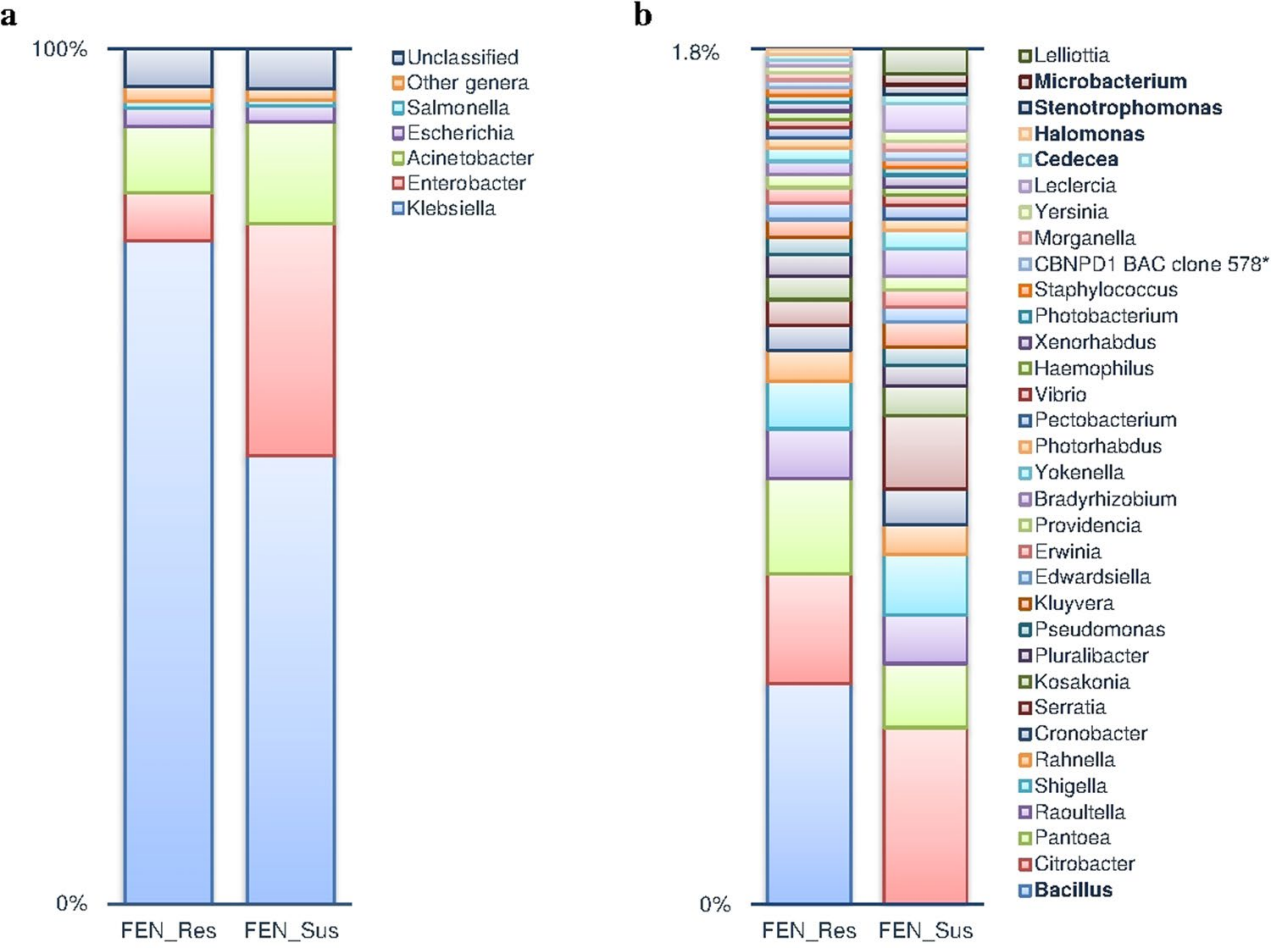
Differential bacterial composition between fenitrothion resistant and susceptible *An. albimanus*. Based on the alignment of sequencing reads (FEN_Res, n = 83,947,332; FEN_Sus, n = 60,444,900) to the NCBI-NR protein database, 37 bacterial genera and one uncultured β-proteobacterium* were identified in fenitrothion resistant and susceptible *An. albimanus* samples. Five genera; *Klebsiella, Enterobacter, Acinetobacter, Escherichia* and *Salmonella* comprised over 93% of identified genera in both samples and are considered as the predominant genera in this study. Plot (**a**) shows the proportions of each predominant genera as well as the proportions of unclassified reads in fenitrothion resistant and susceptible samples. Thirty two bacterial genera comprising the remaining proportion (1.8%) of the microbiota are grouped as ‘other genera’ and expanded in plot (**b**). Bacterial genera that are unique to either resistant or susceptible *An. albimanus* are presented in bold typeface. There were differential abundances of predominant (**a**) and other (**b**) genera between FEN_Res and FEN_Sus, with significant (p < 0.001) enrichment of *Klebsiella* in FEN_Res.

Based on the alignment of sequencing reads to the National Center for Biotechnology Information (NCBI) bacterial non-redundant (NR) protein database and subsequent annotation using the NCBI taxonomy tree, a total of 103 bacterial species and an uncultured β-proteobacterium (CBNPD1 BAC clone 578) were identified in FEN_Res and FEN_Sus. This comprised four phyla, six classes, 12 orders, 13 families, and 37 genera (Fig. 1 and Supplementary Table 1). Thirty-two out of these 37 genera were found in both FEN_Res and FEN_Sus, two (*Bacillus* and *Halomonas*) were unique to FEN_Res, and three (*Stenotrophomonas, Microbacterium*, and *Lelliottia*) were only identified in FEN_Sus (Table 1). The uncultured β-proteobacterium was found in both FEN_Res and FEN_Sus. Proteobacteria was the most abundant bacterial phylum, comprising 99% of reads in each sample. Likewise, Gammaproteobacteria, comprising 99% of reads, was the most predominant bacterial class. At the order level, Enterobacteriales and Pseudomonadales comprised over 98% of reads in each sample, and Enterobacteriaceae and Moraxellaceae comprised 99% of reads per sample at the family level. At the genus level, *Klebsiella, Enterobacter, Acinetobacter, Escherichia*, and *Salmonella* constituted over 93% of reads in each sample, and are considered as the predominant bacterial genera in this study (Fig. 1). The most predominant bacterial species was *Klebsiella pneumoniae*, comprising 74% and 49% of reads in FEN_Res and FEN_Sus, respectively (Supplementary Table 1).

**Table 1:** Differential abundance of identified bacterial genera in fenitrothion resistant and susceptible *An. albimanus*. The table shows the relative abundance (%) of bacterial genera identified in each sample, and the difference in relative abundance of each genera with 95% confidence intervals, calculated using two-sided Fisher’s exact test with Benjamini Hochberg’s false discovery rate p-value corrections. The level of significance was set to p < 0.05. Bacterial genera unique to either sample are presented in bold typeface. The relative abundance of 21 of these bacterial genera was significantly higher in FEN_Res compared to FEN_Sus, with the proportion of *Klebsiella* showing the greatest enrichment (Diff 25.1%, p < 0.0001). Conversely, the proportion of *Enterobacter* was the most reduced in FEN_Res compared to FEN_Sus (Diff −21.5% p < 0.0001).

S/N	Bacterial genera	FEN_Res Rel. abundance (%)	FEN_Sus Rel. abundance (%)	Diff. Rel abundance (%)	95% lower CI	95% upper CI	p-values	Corrected p-values
1	*Klebsiella* ^†,‡^	77.571	52.424	25.148	25.129	25.167	<1E-301	<1E-301
2	***Bacillus*** ^**†**^	**0.456**	**—**	**0.456**	**0.454**	**0.458**	**<1E-301**	**<1E-301**
3	*Escherichia* ^†,‡^	2.239	1.846	0.393	0.387	0.398	<1E-301	<1E-301
4	*Pantoea* ^†,‡^	0.196	0.100	0.096	0.094	0.097	<1E-301	<1E-301
5	*Raoultella* ^†^	0.103	0.076	0.028	0.026	0.029	<1E-301	<1E-301
6	*Salmonella* ^†^	0.738	0.718	0.020	0.016	0.023	1.70E-28	3.82E-28
7	*Rahnella* ^†^	0.065	0.046	0.018	0.018	0.019	1.54E-301	6.04E-301
8	*Pluralibacter* ^♦^	0.044	0.032	0.012	0.011	0.012	3.40E-173	1.23E-172
9	***Halomonas*** ^**±,c**^	**0.011**	**—**	**0.011**	**0.011**	**0.012**	**<1E-301**	**<1E-301**
10	*Edwardsiella* ^†^	0.035	0.024	0.011	0.010	0.012	7.82E-208	2.94E-207
11	*Pseudomonas* ^†,‡^	0.037	0.028	0.009	0.008	0.010	1.37E-124	4.44E-124
12	*Providencia* ^†^	0.027	0.022	0.006	0.005	0.006	3.26E-69	9.28E-69
13	*Erwinia* ^†,‡†,‡^	0.031	0.026	0.005	0.005	0.006	3.80E-51	9.65E-51
14	*Haemophilus* ^†^	0.017	0.012	0.005	0.005	0.006	2.40E-94	7.53E-94
15	*Photorhabdus* ^†^	0.023	0.018	0.005	0.004	0.006	3.75E-60	9.78E-60
16	*Photobacterium* ^♦^	0.016	0.012	0.003	0.003	0.004	6.02E-43	1.49E-42
17	*Shigella* ^†,‡^	0.097	0.094	0.003	0.002	0.004	1.10E-06	1.85E-06
18	*Staphylococcus* ^†^	0.015	0.012	0.003	0.003	0.004	2.53E-36	5.94E-36
19	*Kosakonia* ^♦^	0.049	0.046	0.003	0.002	0.004	5.99E-10	1.10E-09
20	*CBNPD1 BAC clone 578^♦^	0.016	0.013	0.003	0.002	0.003	3.61E-24	7.72E-24
21	*Vibrio* ^†^	0.017	0.016	0.001	0.000	0.001	0.02556166	0.039390101
22	*Morganella* ^†^	0.015	0.015	−0.001	−0.001	0.000	0.02829376	0.042896987
23	*Pectobacterium* ^♦^	0.021	0.022	−0.001	−0.002	−0.001	1.01E-05	1.66E-05
24	*Xenorhabdus* ^†^	0.017	0.019	−0.002	−0.002	−0.001	2.97E-10	5.58E-10
25	*Yokenella* ^±a^	0.026	0.028	−0.002	−0.002	−0.001	2.62E-07	4.56E-07
26	*Yersinia* ^†,‡^	0.014	0.016	−0.002	−0.003	−0.002	7.66E-18	1.56E-17
27	*Cedecea* ^†^	0.012	0.014	−0.002	−0.003	−0.002	3.05E-25	6.66E-25
28	*Cronobacter* ^†^	0.052	0.055	−0.003	−0.004	−0.002	1.03E-10	2.02E-10
29	*Kluyvera* ^†^	0.036	0.039	−0.003	−0.004	−0.002	1.47E-15	2.95E-15
30	***Stenotrophomonas*** ^**†**^	**—**	**0.016**	**−0.016**	**−0.016**	**−0.015**	**<1E-301**	**<1E-301**
31	*Bradyrhizobium* ^†^	0.027	0.044	−0.017	−0.017	−0.016	<1E-301	<1E-301
32	***Microbacterium*** ^**†**^	**—**	**0.017**	**−0.017**	**−0.017**	**−0.016**	**<1E-301**	**<1E-301**
33	*Leclercia* ^♦^	0.013	0.042	−0.029	−0.030	−0.028	<1E-301	<1E-301
34	***Lelliottia*** ^**♦**^	**—**	**0.039**	**−0.039**	**−0.040**	**−0.038**	**<1E-301**	**<1E-301**
35	*Citrobacter* ^†,‡^	0.227	0.276	−0.049	−0.051	−0.047	<1E-301	<1E-301
36	*Serratia* ^†,‡^	0.052	0.115	−0.063	−0.064	−0.061	<1E-301	<1E-301
37	*Acinetobacter* ^†,‡^	7.742	11.913	−4.172	−4.184	−4.159	<1E-301	<1E-301
38	*Enterobacter* ^†,‡^	5.563	27.091	−21.528	−21.543	−21.513	<1E-301	<1E-301
39	Unclassified Bacteria	4.381509301	4.674589901	−0.2930806				

^**†**^Previously identified in *Anopheles*.

^**‡**^Previously identified in Latin American *Anopheles*.

*Uncultured β-proteobacteria.

^**±**^Reported in *Anopheles* for the first time in this study.

^**a**^Only reported in *Aedes* previously.

^**c**^Only reported in *Culex* previously.

^♦^Reported in mosquitoes for the first time in this study.

**Figure 2:**
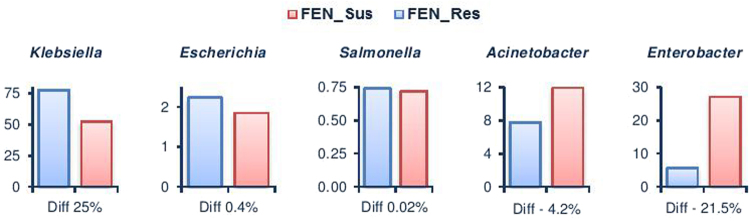
Differential abundance of predominant bacterial genera in fenitrothion resistant and susceptible *An. albimanus*. Plots show the difference in relative abundance (%) of each bacterial genera between fenitrothion resistant and susceptible samples (p < 0.0001).

There were significant differences in the relative abundance of identified bacterial taxa between FEN_Res and FEN_Sus, with lower Simpson’s reciprocal index (a measure of taxonomic richness and evenness) in FEN_Res (1.4) compared to FEN_Sus (2.5). Out of the four bacterial phyla identified in both samples, the relative abundances of Firmicutes and Actinobacteria was significantly (p < 0.0001) higher in FEN_Res compared to FEN_Sus, with a corresponding lower (p < 0.0001) relative abundance of Proteobacteria in FEN_Res. There was no significant difference in the relative abundance (Diff) of Bacteroidetes between both samples (p = 0.43) (Supplementary Table 1). However, since Proteobacteria was the predominant bacterial phylum in both samples (Supplementary Table 1), bacteria belonging to this phylum showed differential compositions between FEN_Res and FEN_Sus at lower taxonomic levels (Supplementary Table 3). The relative abundances of eighteen of the 32 bacterial genera as well as the uncultured β-proteobacterium identified in both samples were significantly (p < 0.001) higher in FEN_Res compared to FEN_Sus (Table 1). These enriched bacterial genera comprised three of the predominant genera; *Klebsiella, Escherichia* and *Salmonella*, with *Klebsiella* being the most enriched (Diff 25.2%, p < 0.0001) in FEN_Res overall (Fig. 2). There was a significantly reduced relative abundance of the remaining 14 genera in FEN_Res (Table 1), with *Enterobacter* showing the greatest reduction (Diff −21.5%, p < 0.0001) (Fig. 2). At the species level, 29 bacterial species were significantly enriched (p < 0.01) in FEN_Res compared to FEN_Sus (Supplementary Table 3), with *Klebsiella pneumoniae* being the most enriched (Diff 24.3%, p < 0.0001). Conversely, the relative abundance of 34 bacterial species was significantly reduced in FEN_Res compared to FEN_Sus (p < 0.001), with *Enterobacter cloacae* showing the greatest reduction (Diff −13.3%, p < 0.0001) (Supplementary Table 3). Out of the remaining 40 bacterial species, seven were unique to FEN_Res, while 33 were only identified in FEN_Sus (Supplementary Table 1).

### Differential abundance of bacterial xenobiotic-degrading enzymes between fenitrothion resistant and susceptible *An. albimanus*, with significant enrichment of OP-degrading carboxylesterases and phosphomonoesterases in resistant *An. albimanus*

A total of 88 bacterial xenobiotic-degrading enzymes were identified in FEN_Res and FEN_Sus based on the Kyoto Encyclopedia of Genes and Genomes (KEGG) annotations of aligned sequencing reads. This comprised all six major classes of enzymes: oxidoreductases, transferases, hydrolases, isomerases, lyases, and ligases, with oxidoreductases, transferases and hydrolases constituting over 80% of identified enzymes in each sample. Within each enzyme class, the most abundant enzymes in both samples were consistent: fumarate reductase (oxidoreductase); glutathione S-transferase and acetyl-CoA C-acetyltransferase (transferase); carboxymethylenebutenolidase (hydrolase); muconate cycloisomerase (isomerase) 4-carboxymuconolactone decarboxylase (lyase); guanosine monophosphate synthase (ligase); and enoyl-CoA isomerase (isomerase) (Supplementary Table 2).

**Figure 3:**
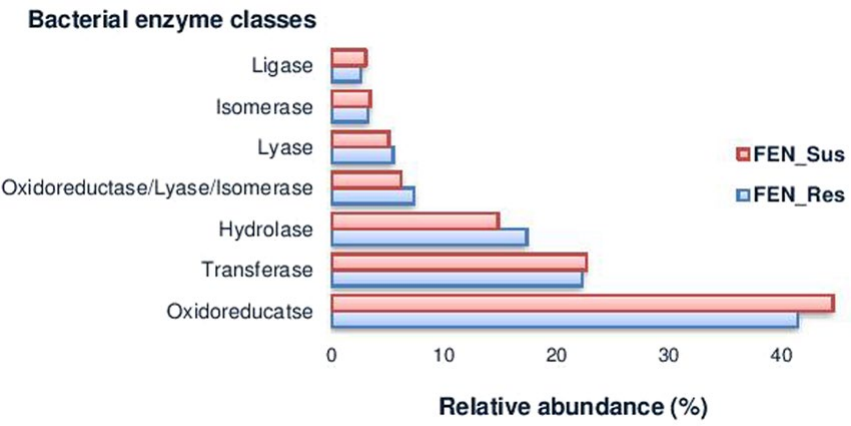
Relative abundance of bacterial enzyme classes associated with xenobiotic degradation pathways in fenitrothion resistant and susceptible *An. albimanus*. Bar plot shows the relative abundance (%) of reads assigned to each enzyme class in FEN_Res (n = 458,614) and FEN_Sus (n = 311,919).

**Figure 4:**
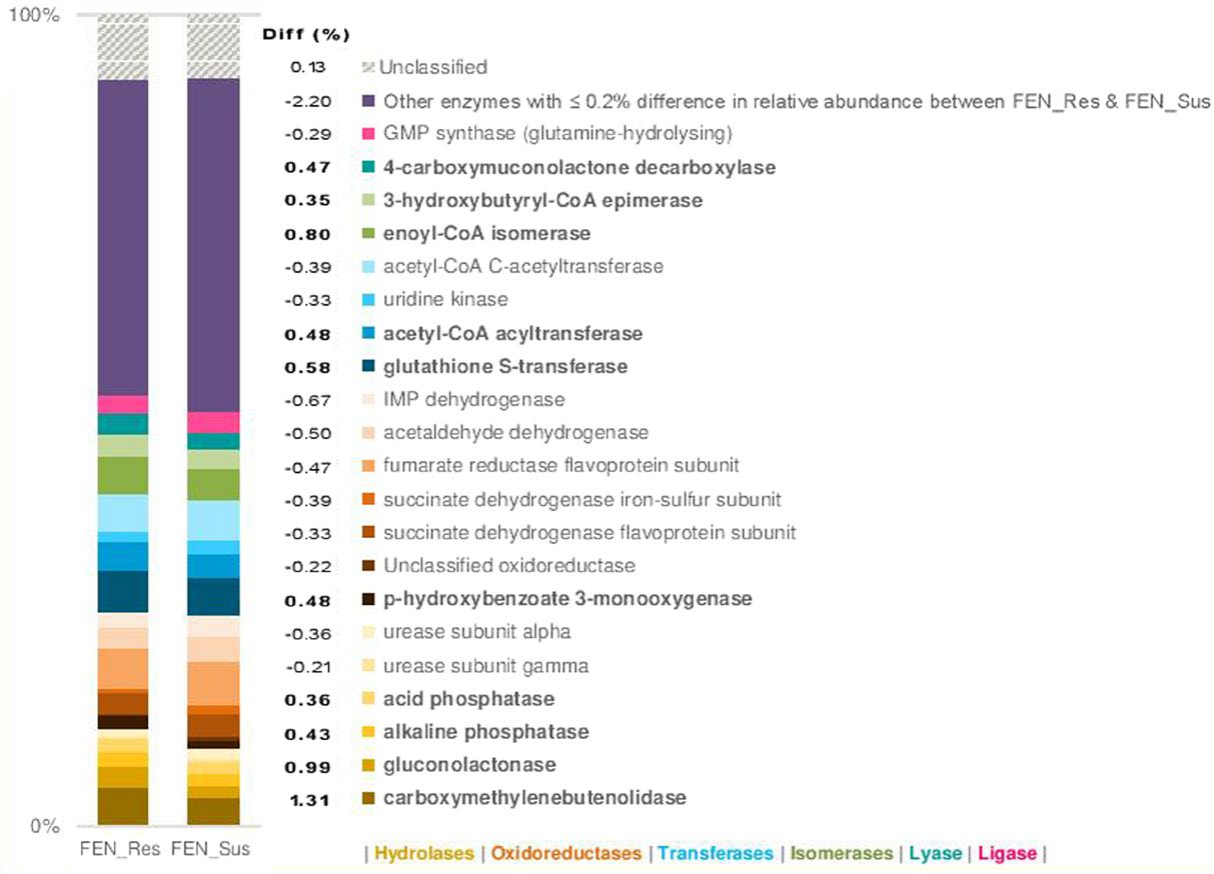
Putative bacterial enzymes involved in microbial xenobiotic degradation in fenitrothion resistant and susceptible *An. albimanus*. The plot shows the proportion (%) of sequencing reads (FEN_Res, n = 458,614; and FEN_Sus, n = 311,919) aligned to bacterial enzymes in the microbial xenobiotic degradation pathway, and the difference in relative abundance of identified enzymes between fenitrothion resistant and susceptible *An. albimanus* (Diff.). Only enzymes with Diff. ≥ 0.2% at p < 0.0001 are shown, and significantly enriched enzymes in FEN_Res are presented in bold typeface.

The relative abundance of hydrolases (Diff 2.55%), isomerases (Diff 1.15%), and lyases (Diff 0.39%) was higher in FEN_Res compared to FEN_Sus. This corresponded to a reduced relative abundance of oxidoreductases (Diff −3.12%), ligases (Diff −0.44%), transferases (Diff −0.34%) and isomerases (Diff −0.22%) in FEN_Res (Fig. 3). Hydrolases, the most enriched enzyme class, comprised of two significantly (p < 0.0001) enriched carboxylesterases; carboxymethylenebutenolidase (Diff 1.31%) and gluconolactonase (Diff 0.99%), and two significantly (p < 0.0001) enriched phosphomonoesterases; alkaline phosphatase (Diff 0.43%) and acid phosphatase (Diff 0.36%) in FEN_Res compared to FEN_Sus, with the carboxylesterases being the most enriched overall (Fig. 4 and Supplementary Table 2). With the exception of ligase, six other enzymes in the remaining classes were also significantly (p < 0.0001) enriched in FEN_Res compared to FEN_Sus: transferases (acetyl-CoA acyltransferase, Diff 0.48% and glutathione S-transferase, Diff 0.58%); lyase (4-carboxymuconolactone decarboxylase, Diff 0.47%); oxidoreductase (p-hydroxybenzoate 3-monooxygenase, Diff 0.48%); and isomerases (3-hydroxybutyryl-CoA epimerase, Diff 0.35%, and enoyl-CoA isomerase, Diff 0.80%) (Fig. 4 and Supplementary Table 2).

### The microbial xenobiotic degradation pathways in fenitrothion resistant *An. albimanus* are comprised of fenitrothion-degrading and other OP-degrading bacterial species that were also confirmed via *in vitro* insecticide treatments

Sequencing reads associated with annotated microbial xenobiotic degradation pathways in FEN_Res mapped to 21 bacterial species belonging to 11 genera (Table 2). Of these, 13 bacterial species belonged to the predominant genera – *Klebsiella, Enterobacter, Acinetobacter, Escherichia*, and *Salmonella*. Bacteria belonging to the genus *Bacillus* were only identified in FEN_Res (Table 2 and Supplementary Table 1). With the exception of *Pluribacter*, each bacterial genus associated with xenobiotic degradation in FEN_Res contained bacterial species that have been documented to degrade OP compounds, including fenitrothion, as well as other classes of pesticides (Table 2). The presence of *B. cereus* and *A. baumannii*, two OP-degrading bacteria identified by WMS in this study (Table 2), was confirmed by PCR following *in vitro* insecticide treatments of the microbiota of FEN_Res.

**Table 2:** Bacterial species associated with microbial xenobiotic degradation in fenitrothion resistant *An. albimanus*. The table lists identified bacterial species, documented OP compounds, and other pesticides metabolized by each associated bacteria.

S/N	*Bacterial species*	OPs metabolized	Other pesticides metabolized	Source
1	*Acinetobacter baumannii*	Fenitrothion, Diazinon, Chlorpyrifos, Malathion, Methyl parathion	Diclofop-methyl	^80,91,92^
2	Acinetobacter calcoaceticus	Chlorpiryfos, Biological phosphorus in activated sludge	—	^93–97^
3	*Acinetobacter pittii*	—	—	^—^
4	*Bacillus anthracis* ^†^	—	—	^—^
5	*Bacillus cereus* ^†^	Dimethoate, Malathion, Chlorpiryfos, Malaoxon, Monocrotophos, Acephate, Phosphonates	DDT, Cypermethrin, Fenvalerate	^98–105^
6	*Bacillus thuringiensis* ^†^	Malathion	Fipronil	^106–108^
7	*Citrobacter koseri*	Glyphosphate, Profenofos	—	^109,110^
8	*Enterobacter aerogenes*	Malathion	Bifenthrin, Fenpropathrin, Cypermethrin, DDT	^103,104,106,111^
9	*Enterobacter asburiae*	Acephate, Glyphosphate	Cypermethrin, Endosulfan, Quizalofop-p-ethyl, Clodinafop, Metribuzin	^109,118,113^
10	*Enterobacter cloacae*	Dimethoate, Glyphosphate	DDT, Endosulfan	^98,103,114–116^
11	*Enterobacter hormaechei*	—	—	^—^
12	*Escherichia coli*	Fenitrothion, Phosphonates	Cypermethrin, DDT, Aldicarb	^103,104,117–119^
13	*Klebsiella oxytoca*	Dimethoate, Chlorpiryfos, Phosphonates	Endosulfan	^120–123^
14	*Klebsiella pneumoniae*	Fensulfothion, Tributyl phosphate, Methyl-parathion, Phosphonates	DDT, Endosulfan, Triazines, Bromoxynil, Imidacloprid	^103,114,123–129^
15	*Klebsiella quasipneumoniae*	—	—	^—^
17	*Klebsiella variicola*	—	—	^—^
18	*Kluyvera cryocrescens*	Phosphonates	—	^123^
19	*Pluralibacter gergoviae*	—	—	^—^
20	*Salmonella enterica*	Phosphonates	—	^130^
21	*Serratia marcescens*	Fenitrothion, Chlorpiryfos, Diazinon, Coumaphos, Parathion, Isazofos, Monocrotophos	—	^100,114,131–133^

^†^Only identified in fenitrothion resistant *An. albimanus*.

## Discussion

Mosquito microbiota has been shown to impact several key host characteristics including growth, nutrition, reproduction, parasite interactions, and vector competence^33^, and the present study provides the first comprehensive characterization of mosquito microbiota in relation to insecticide resistance. The findings presented here show differential composition of the microbiota and its functions between fenitrothion resistant and susceptible *An. albimanus*, with significant enrichment of OP degrading bacteria and putative enzymes in FEN_Res compared to FEN_Sus. Bacteria belonging to the genera *Serratia, Enterobacter, Flavobacterium, Pseudomonas* and *Acinetobacter*^36,37^ have previously been identified in *An. albimanus*. In the present study, 103 bacterial species belonging to 37 bacterial genera (including four of the previously identified genera and unclassified Flavobacteriaceae) were identified, providing a comprehensive update to the composition of *An. albimanus* microbiota. Bacteria belonging to the phylum Proteobacteria were predominant in the present study, indicating that the composition at the phylum level is similar to that of *Anopheles* described previously from Asia^38–42^, Africa^43–46^ and the Americas^36,37,47^. The predominant bacterial genera identified in the present study were *Klebsiella, Enterobacter, Acinetobacter, Escherichia*, and *Salmonella*. These have all been previously identified in *Anopheles*, and all, except *Salmonella*, have previously been documented in Latin American *Anopheles*^48^. The majority (29 out of 37) of all bacterial genera identified in the current study have previously been identified in *Anopheles*^29,35,48^, with 11 of these documented in Latin American *Anopheles*^48^. Out of the eight remaining genera, *Yokenella* and *Halomonas* have so far been reported in *Aedes*^49^ and *Culex*^26^ respectively, while *Kosakonia, Leclercia, Photobacterium, Pluralibacter, Pectobacterium* and *Lelliottia* are documented herein for the first time in *Anopheles*. Two of these newly documented genera have been identified in other insect vectors of human disease, *Pluralibacter* in sandflies (*Phlebotomus chinensis*, a primary vector of leishmaniasis in China)^50^ and *Pectobacterium* in triatomine bugs (*Rhodnius prolixus*, an important vector of Chagas disease in Latin America)^51^. The remaining genera have been identified in insects of agricultural importance: *Kosakonia* in the coffee berry borer (*Hypothenemus hampei*), *Photobacterium* in the date palm borer (*Oryctes Agamemnon*)^52^, *Lelliotia* in the Asian honeybee (*Apis dorsata*)^53^, and *Leclercia* in several classes of agricultural pests^54–57^. Bacterial species within two of these genera have been shown to degrade xenobiotics, such as caffeine by *K. cowanii* within the coffee berry borer^58^, and clorpyrifos ethyl, an OP pesticide, by *L. adecarboxylata* isolated from the fall armyworm (*Spodoptera frugiperda*)^55^.

The lower bacterial diversity observed in FEN_Res compared to FEN_Sus could be a consequence of insecticide exposure, whereby bacteria with the ability to effectively utilize fenitrothion as a nutrient source dominate the microbiota, displacing other bacteria with a resulting decrease in bacterial diversity. Such shifts in microbial composition due to insecticide exposure have previously been documented^59^. For example, the application of fenitrothion to soil in an experimental setting resulted in a significant enrichment of fenitrothion degrading bacteria in the treated soil, from previously undetectable levels to >80% of identified bacteria^21^. Furthermore, the exposure of the diamondback moth, *Plutella xylostella*, to two different organophosphate insecticides resulted in significant enrichment of Lactobacillales in insecticide treated moths^60^. In the present study, *Klebsiella* was significantly enriched in FEN_Res compared to FEN_Sus, with a corresponding reduction in the relative abundance of *Enterobacter*. This was also evident in other genera, as well as at the species level. While this could have occurred as a consequence of insecticide exposure, this may not have been the principal factor due to the short time of insecticide exposure during the bioassays (30 minutes)^14^. This posits the presence of other factors such as a previous selection for OP-degrading bacteria that might have developed alongside resistance to fenitrothion in the mosquitoes. This is exemplified in the diamondback moth, where susceptible and resistant moths from a generation with no insecticide exposure showed differential bacterial compositions. The resistant moths from the unexposed generation showed significant enrichment of Lactobacillales, a bacterial taxa that was also enriched in resistant moths from a different generation that had been exposed to insecticides^60^. These together suggest the enrichment of bacterial taxa with a competitive advantage over other taxa in response to selection pressure. This could also explain finding certain bacterial taxa in one sample and not the other, for instance, the identification of *Bacillus* and *Halomonas* only in FEN_Res, and *Stenotrophomonas, Microbacterium*, and *Lelliottia* only in FEN_Sus. It is also possible that rather than arising from selective pressures, the mere presence of certain bacteria could be mediating insecticide resistance in mosquitoes, and both possibilities merit further exploration.

Other factors such as a mosquito’s physiological status^61^ and age^24^, have been shown to affect the composition of the mosquito microbiota. We recognize that such factors may also be at play in the present study, particularly because wild-caught *An. albimanus* with unknown age and physiological status were utilized^14^. Nonetheless, the findings in this study showed significant enrichment of bacterial carboxylesterases and phosphomonoesterases (Fig. 4) – critical enzyme families involved in bacterial OP-degradation^62–64^ – in FEN_Res compared to FEN_Sus, suggesting that the microbiota in FEN_Res may be involved in OP-degradation. The present study, which reports the first description of putative microbial enzymes associated with xenobiotic degradation in mosquitoes (Supplementary Table 2), detected all major enzyme classes in FEN_Res and FEN_Sus, with oxidoreductases, transferases and hydrolases constituting the majority of identified enzymes (Fig. 3). These three classes of enzymes catalyze the biodegradation of various xenobiotics^65,66^ including OPs, whose catabolism is initiated by hydrolysis – the first and most critical step in OP degradation, which is primarily catalyzed by hydrolases^67^. The most abundant enzyme families (fumarate reductase^68,69^, glutathione S-transferase^70–72^, acetyl-CoA C-acetyltransferase^73,74^, carboxymethylenebutenolidase^75^, muconate cycloisomerase^76^, 4-carboxymuconolactone decarboxylase^77^, guanosine monophosphate synthase^78^, and enoyl-CoA isomerase^79^) within these three classes have all been documented in bacterial xenobiotic degradation. This suggests the potential involvement of *An. albimanus* microbiota in xenobiotic degradation within the mosquito host. Interestingly, hydrolases, the most important class of enzymes involved in bacterial degradation of OPs, were the most significantly enriched in FEN_Res compared to FEN_Sus (Figs 3 and 4). These enriched hydrolases^63,64,67^, along with other enriched enzymes such as glutathione S-transferase^80^ have been documented in bacterial degradation of OPs, further suggesting the involvement of the microbiota in host xenobiotic degradation. The identification of OP-degrading bacterial species, including fenitrothion-degrading species, in association with FEN_Res microbial degradation pathways (Table 2), plus the validation via *in vitro* assays also suggests the involvement of the microbiota in host xenobiotic degradation. It should be noted that although microbial xenobiotic degradation has been studied extensively (particularly in light of increasing interests in bioremediation), the focus has been on cultivable bacteria, which constitute <1% of environmental bacteria^59^. Thus, the xenobiotic degradation profiles of the majority of known bacteria, as well as their corresponding genes and gene products are yet to be characterized. In the present study, all but six of the identified bacterial species associated with microbial xenobiotic degradation in FEN_Res (*A. pittii, B. anthracis*, *E*. *hormaechei*, *K*. *quasipneumoniae*, *K*. *variicola*, and *P*. *gergoviae*) have been documented in OP-metabolism, as well as in the metabolism of other pesticides (Table 2). For these six bacterial species, this may be the first documentation of their association with xenobiotic degradation.

The findings presented here demonstrate differences between the microbiota of FEN_Res and FEN_Sus, as well as associations between the mosquito microbiota and xenobiotic degradation. These initial findings lay the groundwork for future research that will characterize and compare the expression levels of specific microbial genes involved in insecticide degradation, and further elucidate the role of the mosquito microbiota in conferring resistance to insecticides.

## Methods

### Sample collection and determination of resistance profiles

Female *An*. *albimanus* samples collected from La Jota, Tumbes, Peru in 2014, were screened for fenitrothion resistance using the CDC bottle bioassay^81^. Briefly, samples were exposed to bottles coated with one to five times the diagnostic dose (50 µg/bottle) of fenitrothion for 30 mins. These were subsequently removed and classified as susceptible (FEN_Sus), if they were knocked down at the diagnostic dose, or resistant (FEN_Res), if they survived five times the diagnostic dose. The sample collection and bioassay procedures have previously been described in detail^14^. Immediately following the bioassays, samples were stored at −20 °C until processed.

### Preparation of genomic DNA

Pools of 10 (FEN_Sus) and 30 (FEN_Res) whole mosquito samples were processed to digest mosquito host DNA using the MolYsis™ Complete 5 kit (Molzym, Bremen, Germany) following manufacturer’s instructions. We processed available samples as pools to reduce the effect of the variation found in the microbiota among individual mosquitoes of the same species^44^. Prior to host DNA removal, each pool of mosquitoes was surface sterilized by suspending in 70% ethanol and agitating with a vortex mixer for 15–20 seconds, then re-suspending in nuclease free water with agitation for another 15–20 seconds, followed by a final rinse with nuclease free water. Each pool was subsequently suspended in 50 µL of MolYsis chaotrophic buffer, CM, and homogenized to begin the host DNA removal process.

Following the removal of mosquito DNA, genomic DNA was extracted from FEN_Sus using DNeasy Blood and Tissue Kit (QIAGEN), and from FEN_Res using MolYsis™ Complete 5 kit, per manufacturers’ instructions. In processing FEN_Sus, which had less mosquitoes/pool, the DNeasy blood and Tissue Kit was substituted for MolYsis to optimize the yield of metagenomic DNA. Negative controls for the extractions (tubes without mosquitoes) were included with each extraction process. For all extractions, DNA was eluted to a final volume of 60 µL and stored in −20 °C until library preparation and sequencing. Prior to library preparation and sequencing, each extraction product was tested for the presence of the 16S rRNA gene using the HDA1 and HDA2 primers^23^. Both DNA samples from FEN_Res and FEN_Sus were positive, while both extraction controls were negative.

### Library preparation and whole metagenome sequencing

Genomic DNA was sheared to a mean size of 600 bp using a Covaris LE220 focused ultrasonicator (Covaris Inc., Woburn, MA). DNA fragments were cleaned with Ampure (Beckman Coulter Inc., Indianapolis, IN) and used to prepare dual-indexed sequencing libraries using the NEBNext Ultra library prep reagents (New England Biolabs Inc., Ipswich, MA), and barcoding indices synthesized at the CDC Biotechnology Core Facility. Libraries were analyzed for size and concentration, pooled and denatured for loading onto flowcells for cluster generation. Sequencing was performed on an Illumina HiSeq2500 platform using HiSeq Rapid SBS 250 × 250 cycle paired-end sequencing kits. Each library pool was loaded onto both lanes of the HiSeq Rapid flowcell, and on completion, sequence reads were filtered for read quality, basecalled and demultiplexed using Casava (v1.8.2). There was no significant difference in resulting reads from either lane of the flowcell. Thus, only outcomes from one lane of the flowcell are reported.

### Sequencing data quality control and removal of mosquito genome

Demultiplexed reads from each sample were examined for quality using FastQC v0.11.5^82^. Adapters and low quality reads were removed using Trimmomatic v0.35^83^. A custom adapter file containing TruSeq universal and index primers, as well as each primers’ reverse complement, was used with the ILLUMINACLIP command in Trimmomatic to remove adapters. Next, the first 14 and last five bases were removed from each read, and the resulting reads were scanned using a sliding window of four nucleotides. Nucleotides within each sliding window were removed until the average Phred score across the window was >20. Finally, sequences along with their mate-pairs with length less than 60 bp were removed.

The quality trimmed sequences for each sample were aligned to the host reference genome (*An*. *albimanus* STECLA strain, AalbS2 assembly^84^) using the BWA-MEM algorithm of the Burrows-Wheeler aligner (BWA v0.77)^85^ with default settings for paired-end reads (BWA alignments have been deposited in the NCBI Sequence Read Archive, SRA; SRR5630719 and SRR5630720). Reads that aligned to *An. albimanus* genome were removed, and resulting non-host reads were checked for quality with FastQC and used in downstream analysis.

### Taxonomic and functional annotations

To assess taxonomic and functional composition, non-host reads were aligned to the NCBI-NR database (downloaded June, 2016) using the BLASTx algorithm in DIAMOND v0.8.6v^86^, with the following adjusted parameters –e 0.00001,–top 3, –f 6, and –c 1. Each read pair was analyzed separately, then subsequent aligned reads were merged, imported into the MEtaGenome ANalyzer (MEGAN) v.6.5.7^87^ using the paired-read mode, and parsed using default parameters of the lowest common ancestor (LCA) algorithm. NCBI taxonomy (prot-acc2taxid-August2016.bin) and the Kyoto Encyclopedia of Genes and Genomes, KEGG (gi2kegg-Feb2015X.bin) mapping files were used to map aligned reads to the NCBI taxonomy tree and KEGG pathways, respectively. One final check for host genome contamination was performed at this stage, and reads that mapped to the Eukaryota domain were removed from downstream analysis. To identify bacteria associated with bacterial xenobiotic degradation pathways, reads that mapped to the KEGG bacterial xenobiotic degradation pathways were extracted and processed for taxonomic annotation as described above.

### Comparisons and statistical analysis

A single MEGAN comparison file was generated using both FEN_Res and FEN_Sus individual MEGAN files. Simpson’s reciprocal indices were calculated for each sample using MEGAN, to determine species diversity (the number of identified bacteria taxa) and evenness (the relative abundance of each identified taxa) within each sample. Taxonomic profiles at each level of classification and corresponding read abundances were exported from the comparison file into the Statistical Analysis of Metagenomic Profiles (STAMP) software v2.1.3^88^. Likewise, functional (KEGG) profiles were exported into STAMP for statistical analysis. For each sample, the relative abundance of reads assigned to each identified taxon/function were calculated. The resulting relative abundances were compared between samples using two-sided Fisher’s exact test with Benjamini-Hochberg False Discovery Rate (FDR) correction. The level of significance was set to *p* < 0.05, and any taxa/function with < 100 assigned reads in both samples were grouped as unclassified.

### Identification of OP-degrading bacteria in *in vitro* insecticide treatments of *An. albimanus* microbiota

The microbiota of mosquitoes that survived 10 times the diagnostic dose of fenitrothion^81^ from the same mosquito population were cultured on insecticide treated bacterial media. Three pools of three female mosquitoes each were surface sterilized as described above and homogenized in nuclease free ultra-purified water. One hundred microliters of each homogenate was spread on Luria-Bertani (LB) agar plates that were surface-treated with either 1 mL of the diagnostic dose (50 µg/mL) or five times the diagnostic dose of fenitrothion and incubated overnight at 37 ± 0.5 °C. Untreated LB agar plates and plates treated with 1 mL of absolute ethanol (solvent) were included as controls. Following incubation, bacterial colonies were collected for DNA extraction. Genomic DNA was extracted from bacterial colonies using the Extracta™ DNA Prep for PCR kit (Quanta BioSciences, USA) following manufacturer’s instructions. Using PCR, bacterial genomic DNA was screened for two bacterial species/groups known to degrade organophosphate insecticides including fenitrothion – *A*. *baumannii*, and *B*. *cereus* – that were also associated with putative microbial xenobiotic degradation pathways in FEN_Res. Following a previously described method^89^ with slight modifications, the PCR for *A. baumannii* identification was conducted using *A. baumannii* specific primers P-Ab-ITSF (CATTATCACGGTAATTAGTG) and P-Ab-ITSB (AGAGCACTGTGCACTTAAG), along with internal control primers P-rA1 (CCTGAATCTTCTGGTAAAAC) and P-rA2 (GTTTCTGGGCTGCCAAACATTAC). The specific primer pair (P-Ab-ITSF and P-Ab-ITSB) amplifies a 208 bp fragment of the ITS region in *A. baumannii*, while the internal control primers (P-rA1 and P-rA2) target a 425 bp region of the recA gene in all *Acinetobacter* species. To screen for *B. cereus*, the BCFomp1 (ATCGCCTCGTTGGATGACGA) and BCRomp1 (CTGCATATCCTACCGCAGCTA) primer set^89^ which targets a 575 bp region of the motB gene in *B*. *cereus* was used. PCR was performed in a total reaction volume of 20 µL (*A. baumannii*) and 25 µL (*B. cereus*), each containing 60–160ng/µL DNA template, 1 µM of each respective primer, 12.5 µL of 2 × KAPA HiFi HotStart PCR mix (Roche, Switzerland), and PCR grade water to final volume. Reactions were conducted using a T100™ Thermal Cycler (Bio-Rad, USA) following the previously described conditions^89,90^.

### Data Accessibility

The WMS reads obtained from this study (SRP108310) have been deposited in NCBI under the BioProject PRJNA388280.

## Electronic supplementary material

Supplementary Information
