# Supplementary material for: Whole metagenome sequencing reveals links between mosquito microbiota and insecticide resistance in malaria vectors
Source: Sci Rep. 2018 Feb 1;8:2084. doi: 10.1038/s41598-018-20367-4 (PMC5794770; doi:10.1038/s41598-018-20367-4)
Supplement: Supplementary file 1 — Supplementary Information [file 41598_2018_20367_MOESM1_ESM.pdf]

Supplementary Information for, 'Whole metagenome sequencing reveals links between mosquito microbiota and insecticide resistance in malaria vectors'

Authors: Nsa Dada, Mili Sheth, Kelly Liebman, Jesus Pinto and Audrey Lenhart

Supplementary Table 1. Comparative statistics of all bacteria taxa identified in FEN_Res and FEN_Sus.

S/N	Bacterial species	FEN_Res no. Reads	FEN_Res Rel. abundance (%)	FEN_Sus no. reads	FEN_Sus Rel. abundance (%)	Diff. Rel abundance (%)	95% lower CI	95% upper CI	p-values	Corrected p-values
1	*[Enterobacter] aerogenes*	155090	0.300543511	81044	0.204298502	0.09624501	0.094194422	0.0982956	<1E-301	<1E-301
2	*Acinetobacter baumannii*	1100441	2.132506299	1247329	3.144309828	-1.011803529	-1.01851378	-1.005093	<1E-301	<1E-301
3	*Acinetobacter calcoaceticus*	27313	0.052928912	36234	0.091339913	-0.038411001	-0.03954127	-0.037281	<1E-301	<1E-301
4	*Acinetobacter haemolyticus*	0	0	4355	0.010978234	-0.010978234	-0.01130427	-0.010652	<1E-301	<1E-301
5	*Acinetobacter johnsonii*	29309	0.056796891	9567	0.024116823	0.032680068	0.031870098	0.03349	<1E-301	<1E-301
6	*Acinetobacter junii*	0	0	4672	0.011777338	-0.011777338	-0.01211503	-0.01144	<1E-301	<1E-301
7	*Acinetobacter nosocomialis*	5382	0.01042959	18274	0.046065727	-0.035636137	-0.03635968	-0.034913	<1E-301	<1E-301
8	*Acinetobacter oleivorans*	5276	0.010224177	6780	0.017091257	-0.00686708	-0.00735859	-0.006376	4.12E-174	9.16E-174
9	*Acinetobacter pittii*	1660095	3.2170403	2049567	5.16661896	-1.94957866	-1.95798252	-1.941175	<1E-301	<1E-301
10	*Acinetobacter seifertii*	0	0	5450	0.013738547	-0.013738547	-0.01410327	-0.013374	<1E-301	<1E-301
11	*Acinetobacter soli*	6031	0.011687265	18489	0.046607707	-0.034920442	-0.03565401	-0.034187	<1E-301	<1E-301
12	*Acinetobacter sp. ATCC 27244*	0	0	4390	0.011066463	-0.011066463	-0.0113938	-0.010739	<1E-301	<1E-301
13	*Acinetobacter sp. LMB-5*	0	0	5734	0.014454464	-0.014454464	-0.01482857	-0.01408	<1E-301	<1E-301
14	*Acinetobacter sp. NIPH 542*	0	0	4210	0.010612713	-0.010612713	-0.01093327	-0.010292	<1E-301	<1E-301
15	*Acinetobacter sp. P8-3-8*	10295	0.019950322	18669	0.047061457	-0.027111135	-0.02788831	-0.026334	<1E-301	<1E-301
16	*Acinetobacter sp. WC-136*	0	0	5631	0.014194818	-0.014194818	-0.01456555	-0.013824	<1E-301	<1E-301
17	*Acinetobacter sp. WC-141*	6233	0.012078714	7940	0.020015425	-0.007936711	-0.00846934	-0.007404	1.45E-197	3.26E-197
18	*Acinetobacter ursingii*	0	0	4185	0.010549692	-0.010549692	-0.0108693	-0.01023	<1E-301	<1E-301
19	*Bacillus anthracis*	63791	0.123618358	0	0	0.123618358	0.122659658	0.1245771	<1E-301	<1E-301
20	*Bacillus cereus*	40565	0.078609501	0	0	0.078609501	0.077844827	0.0793742	<1E-301	<1E-301
21	*Bacillus thuringiensis*	6076	0.011774469	0	0	0.011774469	0.011478426	0.0120705	<1E-301	<1E-301
22	*Bradyrhizobium sp. DFCI-1*	0	0	5932	0.01495359	-0.01495359	-0.01533409	-0.014573	<1E-301	<1E-301

S/N	Bacterial species	FEN_Res no. Reads	FEN_Res Rel. abundance (%)	FEN_Sus no. reads	FEN_Sus Rel. abundance (%)	Diff. Rel abundance (%)	95% lower CI	95% upper CI	p-values	Corrected p-values
**23**	*Citrobacter freundii*	15605	0.030240386	15671	0.039503996	-0.009263609	-0.01004299	-0.008484	3.84E-123	8.12E-123
**24**	*Citrobacter koseri*	24288	0.04706687	28707	0.072365593	-0.025298723	-0.02632365	-0.024274	<1E-301	<1E-301
**25**	*Citrobacter sp. 30_2*	9886	0.019157735	9666	0.024366385	-0.00520865	-0.00582386	-0.004593	3.08E-63	5.84E-63
**26**	*Citrobacter sp. BIDMC107*	13541	0.026240632	7679	0.019357487	0.006883145	0.006264513	0.0075018	4.67E-103	9.60E-103
**27**	*Citrobacter sp. BIDMC108*	8311	0.016105598	0	0	0.016105598	0.015759369	0.0164518	<1E-301	<1E-301
**28**	*Citrobacter sp. MGH99*	5978	0.011584558	0	0	0.011584558	0.011290912	0.0118782	<1E-301	<1E-301
**29**	*Citrobacter youngae*	10713	0.02076035	8713	0.02196403	-0.00120368	-0.00180962	-0.000598	9.57E-05	0.0001381
**30**	*Cronobacter sakazakii*	23829	0.046177389	19382	0.048858812	-0.002681423	-0.00358503	-0.001778	5.56E-09	8.73E-09
**31**	*Edwardsiella ictaluri*	13954	0.027040971	6608	0.016657674	0.010383297	0.009781196	0.0109854	1.98E-242	4.75E-242
**32**	*Enterobacter asburiae*	147290	0.285428163	547968	1.381336574	-1.095908411	-1.09982126	-1.091996	<1E-301	<1E-301
**33**	*Enterobacter cancerogenus*	16645	0.032255766	64670	0.163022359	-0.130766593	-0.13211423	-0.129419	<1E-301	<1E-301
**34**	*Enterobacter cloacae*	1422587	2.756781816	6358155	16.02785572	-13.27107391	-13.2833331	-13.25881	<1E-301	<1E-301
**35**	*Enterobacter cloacae complex 'Hoffmann cluster IV'*	38588	0.074778342	114397	0.288375891	-0.213597549	-0.21542532	-0.21177	<1E-301	<1E-301
**36**	*Enterobacter cloacae complex sp. GN04787*	7479	0.014493294	45530	0.114773589	-0.100280296	-0.10138394	-0.099177	<1E-301	<1E-301
**37**	*Enterobacter cloacae complex sp. GN06232*	0	0	7942	0.020020467	-0.020020467	-0.02046073	-0.01958	<1E-301	<1E-301
**38**	*Enterobacter hormaechei*	148495	0.28776329	265151	0.668401757	-0.380638467	-0.38356513	-0.377712	<1E-301	<1E-301
**39**	*Enterobacter kobei*	9103	0.017640387	48732	0.122845301	-0.105204914	-0.10635358	-0.104056	<1E-301	<1E-301
**40**	*Enterobacter ludwigii*	0	0	13678	0.034479973	-0.034479973	-0.03505771	-0.033902	<1E-301	<1E-301
**41**	*Enterobacter mori*	0	0	22744	0.057333857	-0.057333857	-0.05807876	-0.056589	<1E-301	<1E-301
**42**	*Enterobacter sp. 35669*	19734	0.038241832	114220	0.287929703	-0.249687871	-0.25143852	-0.247937	<1E-301	<1E-301
**43**	*Enterobacter sp. 35699*	0	0	6858	0.017287882	-0.017287882	-0.017697	-0.016879	<1E-301	<1E-301
**44**	*Enterobacter sp. 50588862*	10746	0.020824299	72147	0.181870638	-0.161046339	-0.16242944	-0.159663	<1E-301	<1E-301
**45**	*Enterobacter sp. 50858885*	0	0	5498	0.013859547	-0.013859547	-0.01422587	-0.013493	<1E-301	<1E-301
**46**	*Enterobacter sp. 638*	7004	0.013572808	13689	0.034507702	-0.020934894	-0.02159449	-0.020275	<1E-301	<1E-301
**47**	*Enterobacter sp. BIDMC 29*	13222	0.025622453	74261	0.187199682	-0.161577229	-0.16299147	-0.160163	<1E-301	<1E-301
**48**	*Enterobacter sp. BIDMC92*	0	0	8212	0.020701092	-0.020701092	-0.02114878	-0.020253	<1E-301	<1E-301
**49**	*Enterobacter sp. DC1*	0	0	24267	0.061173088	-0.061173088	-0.06194252	-0.060404	<1E-301	<1E-301
**50**	*Enterobacter sp. DC3*	0	0	11154	0.028117387	-0.028117387	-0.02863912	-0.027596	<1E-301	<1E-301

S/N	Bacterial species	FEN_Res no. Reads	FEN_Res Rel. abundance (%)	FEN_Sus no. reads	FEN_Sus Rel. abundance (%)	Diff. Rel abundance (%)	95% lower CI	95% upper CI	p-values	Corrected p-values
**51**	*Enterobacter sp. EGD-HP1*	12555	0.024329897	50840	0.12815922	-0.103829323	-0.10502118	-0.102637	<1E-301	<1E-301
**52**	*Enterobacter sp. FY-07*	47291	0.091643582	44552	0.112308213	-0.020664631	-0.02199426	-0.019335	1.03E-207	2.34E-207
**53**	*Enterobacter sp. GN02283*	0	0	26007	0.06555934	-0.06555934	-0.06635586	-0.064763	<1E-301	<1E-301
**54**	*Enterobacter sp. GN02548*	0	0	22104	0.055720523	-0.055720523	-0.05645488	-0.054986	<1E-301	<1E-301
**55**	*Enterobacter sp. GN03164*	0	0	4104	0.010345504	-0.010345504	-0.010662	-0.010029	<1E-301	<1E-301
**56**	*Enterobacter sp. MGH 14*	0	0	4314	0.01087488	-0.01087488	-0.01119938	-0.01055	<1E-301	<1E-301
**57**	*Enterobacter sp. MGH 23*	0	0	6458	0.016279548	-0.016279548	-0.01667656	-0.015883	<1E-301	<1E-301
**58**	*Enterobacter sp. MGH 24*	0	0	4040	0.010184171	-0.010184171	-0.01049819	-0.00987	<1E-301	<1E-301
**59**	*Enterobacter sp. MGH 25*	0	0	4075	0.0102724	-0.0102724	-0.01058778	-0.009957	<1E-301	<1E-301
**60**	*Enterobacter sp. MT20*	0	0	11705	0.029506366	-0.029506366	-0.03004082	-0.028972	<1E-301	<1E-301
**61**	*Enterobacter sp. SENG-6*	0	0	4420	0.011142088	-0.011142088	-0.01147055	-0.010814	<1E-301	<1E-301
**62**	*Enterobacter sp. T1-1*	0	0	7825	0.019725529	-0.019725529	-0.02016254	-0.019289	<1E-301	<1E-301
**63**	*Enterobacter sp. UCD-UG_FMILLET*	0	0	22395	0.056454086	-0.056454086	-0.05719326	-0.055715	<1E-301	<1E-301
**64**	*Enterobacter xiangfangensis*	0	0	12777	0.032208701	-0.032208701	-0.03276709	-0.03165	<1E-301	<1E-301
**65**	*Erwinia sp. 9145*	8431	0.016338141	5394	0.013597381	0.002740761	0.002237511	0.003244	3.19E-26	5.52E-26
**66**	*Escherichia coli*	1142366	2.213751297	717662	1.809107044	0.404644253	0.398872188	0.4104163	<1E-301	<1E-301
**67**	*Halomonas titanicae*	5770	0.011181482	0	0	0.011181482	0.010892989	0.01147	<1E-301	<1E-301
**68**	*Klebsiella michiganensis*	50408	0.097683908	30699	0.077387095	0.020296813	0.019082201	0.0215114	4.38E-231	1.04E-230
**69**	*Klebsiella oxytoca*	137884	0.267200603	79836	0.201253334	0.065947269	0.063965165	0.0679294	<1E-301	<1E-301
**70**	*Klebsiella pneumoniae*	38277478	74.17659188	19799625	49.91157543	24.26501645	24.24540306	24.28463	<1E-301	<1E-301
**71**	*Klebsiella quasipneumoniae*	44095	0.085450165	18985	0.047858041	0.037592124	0.036543894	0.0386404	<1E-301	<1E-301
**72**	*Klebsiella sp. 1_1_55*	65858	0.127623925	36301	0.091508809	0.036115116	0.034760797	0.0374694	<1E-301	<1E-301
**73**	*Klebsiella sp. 10982*	12667	0.024546938	6055	0.015263652	0.009283286	0.008708417	0.0098582	5.93E-213	1.36E-212
**74**	*Klebsiella sp. 4_1_44FAA*	20764	0.040237833	11057	0.027872866	0.012364967	0.011610476	0.0131195	1.66E-220	3.85E-220
**75**	*Klebsiella sp. AS10*	7265	0.014078591	4816	0.012140338	0.001938252	0.001466724	0.0024098	1.19E-15	1.93E-15
**76**	*Klebsiella sp. KTE92*	6488	0.012572869	4086	0.010300129	0.00227274	0.001833062	0.0027124	9.85E-24	1.64E-23
**77**	*Klebsiella sp. MS 92-3*	332044	0.643456507	185449	0.467486215	0.175970291	0.172926439	0.1790141	<1E-301	<1E-301
**78**	*Klebsiella sp. OBRC7*	16823	0.032600706	6134	0.015462798	0.017137908	0.016511552	0.0177643	<1E-301	<1E-301
**79**	*Klebsiella variicola*	120054	0.232648467	67184	0.169359737	0.06328873	0.061454307	0.0651232	<1E-301	<1E-301
**80**	*Kluyvera cryocrescens*	11908	0.023076099	10169	0.025634365	-0.002558266	-0.00320628	-0.00191	7.52E-15	1.21E-14

S/N	Bacterial species	FEN_Res no. Reads	FEN_Res Rel. abundance (%)	FEN_Sus no. reads	FEN_Sus Rel. abundance (%)	Diff. Rel abundance (%)	95% lower CI	95% upper CI	p-values	Corrected p-values
**81**	*Kosakonia radicincitans*	23526	0.045590216	15693	0.039559454	0.006030762	0.005180963	0.0068806	1.76E-43	3.26E-43
**82**	*Leclercia adecarboxylata*	6678	0.012941064	16178	0.040782059	-0.027840995	-0.02854177	-0.02714	<1E-301	<1E-301
**83**	*Lelliottia amnigena*	0	0	15489	0.039045204	-0.039045204	-0.03965998	-0.03843	<1E-301	<1E-301
**84**	*Microbacterium sp. SUBG005*	0	0	6665	0.016801361	-0.016801361	-0.01720469	-0.016398	<1E-301	<1E-301
**85**	*Morganella morganii*	7522	0.014576622	6009	0.015147694	-0.000571072	-0.0010762	-6.59E-05	0.02642035	0.0362241
**86**	*Pantoea sp. FF5*	8214	0.015917625	0	0	0.015917625	0.015573422	0.0162618	<1E-301	<1E-301
**87**	*Pectobacterium carotovorum*	5958	0.011545801	5575	0.014053652	-0.002507851	-0.00297903	-0.002037	6.40E-26	1.09E-25
**88**	*Photobacterium sp. SKA34*	7116	0.013789849	4479	0.011290817	0.002499031	0.002038635	0.0029594	5.24E-26	8.99E-26
**89**	*Photorhabdus luminescens*	11191	0.021686649	6552	0.016516507	0.005170142	0.004603292	0.005737	5.32E-70	1.03E-69
**90**	*Pluralibacter gergoviae*	22694	0.043977912	12853	0.032400284	0.011577628	0.010777072	0.0123782	8.95E-173	1.97E-172
**91**	*Providencia stuartii*	7474	0.014483604	5220	0.013158755	0.001324849	0.000839862	0.0018098	9.93E-08	1.51E-07
**92**	*Rahnella aquatilis*	33019	0.06398637	18039	0.045473331	0.018513039	0.01755587	0.0194702	9.65E-307	2.36E-306
**93**	*Raoultella ornithinolytica*	40011	0.077535924	23055	0.058117837	0.019418087	0.018350747	0.0204854	1.62E-272	3.93E-272
**94**	*Raoultella planticola*	7691	0.014904121	4418	0.011137046	0.003767075	0.003299348	0.0042348	6.25E-55	1.17E-54
**95**	*Salmonella enterica*	378341	0.733173851	281584	0.709826628	0.023347223	0.019848245	0.0268462	5.66E-39	1.03E-38
**96**	*Serratia marcescens*	15447	0.029934203	30610	0.07716274	-0.047228537	-0.04821312	-0.046244	<1E-301	<1E-301
**97**	*Shigella boydii*	0	0	4062	0.010239629	-0.010239629	-0.01055451	-0.009925	<1E-301	<1E-301
**98**	*Shigella dysenteriae*	7057	0.013675515	5893	0.014855277	-0.001179762	-0.00167537	-0.000684	2.86E-06	4.24E-06
**99**	*Shigella flexneri*	12842	0.024886065	9757	0.024595781	0.000290284	-0.00036035	0.0009409	0.38301959	0.5030016
**100**	*Shigella sonnei*	23842	0.046202582	16459	0.041490413	0.004712169	0.003848805	0.0055755	1.81E-26	3.16E-26
**101**	*Stenotrophomonas maltophilia*	0	0	4859	0.012248734	-0.012248734	-0.01259312	-0.011904	<1E-301	<1E-301
**102**	*Xenorhabdus cabanillasii*	7157	0.013869301	6129	0.015450194	-0.001580893	-0.00208371	-0.001078	6.07E-10	9.58E-10
**103**	*Yokenella regensburgei*	13422	0.026010026	11025	0.027792199	-0.001782173	-0.00246234	-0.001102	2.62E-07	3.94E-07
**104**	*uncultured beta proteobacterium CBNPD1 BAC clone 578*	8263	0.01601258	5320	0.013410839	0.002601741	0.002102711	0.0031008	3.61E-24	6.06E-24
**105**	Unclassified Acinetobacter	1144506	2.217898328	1274406	3.21256646	-0.994668132	-1.00146919	-0.987867	<1E-301	<1E-301
**106**	Unclassified Actinobacteria	11020	0.021355274	1805	0.004550106	0.016805168	0.016354614	0.0172557	<1E-301	<1E-301
**107**	Unclassified Actinobacteria	413	0.000800338	31	7.81E-05	0.000722192	0.00064025	0.0008041	3.44E-66	6.57E-66
**108**	Unclassified Alphaproteobacteria	3094	0.005995755	2473	0.006234023	-0.000238269	-0.0005623	8.58E-05	0.15184486	0.2030808

S/N	Bacterial species	FEN_Res no. Reads	FEN_Res Rel. abundance (%)	FEN_Sus no. reads	FEN_Sus Rel. abundance (%)	Diff. Rel abundance (%)	95% lower CI	95% upper CI	p-values	Corrected p-values
109	Unclassified Bacillaceae	558	0.001081329	0	0	0.001081329	0.000991609	0.001171	9.72E-139	2.10E-138
110	Unclassified Bacillales	1110	0.00215103	1361	0.003430856	-0.001279825	-0.00150171	-0.001058	5.36E-31	9.51E-31
111	Unclassified Bacilli	2698	0.00522836	134	0.000337792	0.004890568	0.004685166	0.005096	<1E-301	<1E-301
112	Unclassified Bacillus	124807	0.24185914	0	0	0.24185914	0.240518953	0.2431993	<1E-301	<1E-301
113	Unclassified Bacteroidetes	5888	0.01141015	386	0.000973042	0.010437108	0.010129939	0.0107443	<1E-301	<1E-301
114	Unclassified Betaproteobacteria	738	0.001430145	602	0.001517542	-8.74E-05	-0.00024659	7.18E-05	0.28262625	0.3734092
115	Unclassified Bradyrhizobiaceae	281	0.00054454	329	0.000829355	-0.000284814	-0.00039475	-0.000175	2.42E-07	3.66E-07
116	Unclassified Bradyrhizobium	13940	0.027013841	11349	0.028608949	-0.001595109	-0.00228649	-0.000904	5.91E-06	8.70E-06
117	Unclassified Burkholderiales	5567	0.010788095	4399	0.01108915	-0.000301055	-0.00073427	0.0001322	0.17255564	0.2293727
118	Unclassified Cedecea	6042	0.011708581	5632	0.014197339	-0.002488758	-0.00296269	-0.002015	3.05E-25	5.15E-25
119	Unclassified Citrobacter	28689	0.055595414	39078	0.098509166	-0.042913752	-0.04408278	-0.041745	<1E-301	<1E-301
120	Unclassified Cronobacter	3138	0.006081021	2604	0.006564253	-0.000483232	-0.00081312	-0.000153	0.00403345	0.0057097
121	Unclassified Edwardsiella	4052	0.00785223	2816	0.00709867	0.00075356	0.000396929	0.0011102	3.88E-05	5.63E-05
122	Unclassified Enterobacter	969964	1.879659464	2702083	6.811503727	-4.931844263	-4.94051589	-4.923173	<1E-301	<1E-301
123	Unclassified Enterobacteriaceae	2016336	3.90738733	1692643	4.266872669	-0.359485339	-0.3677016	-0.351269	<1E-301	<1E-301
124	Unclassified Enterobacteriales	1	1.94E-06	0	0	1.94E-06	-1.86E-06	5.74E-06	0.99999995	1.2674418
125	Unclassified Erwinia	7685	0.014892494	4863	0.012258818	0.002633676	0.00215457	0.0031128	1.16E-26	2.04E-26
126	Unclassified Escherichia	12931	0.025058535	14688	0.037026015	-0.011967481	-0.01270566	-0.011229	2.00E-230	4.68E-230
127	Unclassified Firmicutes	4159	0.008059581	2258	0.005692044	0.002367537	0.00202826	0.0027068	1.72E-41	3.15E-41
128	Unclassified Flavobacteriaceae	0	0	4210	0.010612713	-0.010612713	-0.01093327	-0.010292	<1E-301	<1E-301
129	Unclassified Flavobacteriales	0	0	1	2.52E-06	-2.52E-06	-7.46E-06	2.42E-06	0.4346256	0.5673556
130	Unclassified Gammaproteobacteria	70864	0.137324878	48595	0.122499947	0.014824932	0.013339781	0.0163101	1.92E-84	3.76E-84
131	Unclassified Haemophilus	8778	0.017010581	4674	0.01178238	0.005228201	0.004737596	0.0057188	2.40E-94	4.81E-94
132	Unclassified Halomonadaceae	8	1.55E-05	0	0	1.55E-05	4.76E-06	2.62E-05	0.01171299	0.0163682
133	Unclassified Halomonas	48	9.30E-05	0	0	9.30E-05	6.67E-05	0.0001193	1.47E-12	2.35E-12
134	Unclassified Intrasporangiaceae	5570	0.010793909	0	0	0.010793909	0.010510459	0.0110774	<1E-301	<1E-301
135	Unclassified Klebsiella	782408	1.516201221	464850	1.171809862	0.344391359	0.339665867	0.3491169	<1E-301	<1E-301
136	Unclassified Kluyvera	6477	0.012551553	5253	0.013241943	-0.00069039	-0.00116118	-0.00022	0.00400136	0.0057013
137	Unclassified Kosakonia	1641	0.003180037	2525	0.006365107	-0.00318507	-0.00347714	-0.002893	2.74E-109	5.75E-109
138	Unclassified Lactobacillales	5957	0.011543863	4075	0.0102724	0.001271463	0.000840894	0.001702	8.82E-09	1.37E-08

S/N	Bacterial species	FEN_Res no. Reads	FEN_Res Rel. abundance (%)	FEN_Sus no. reads	FEN_Sus Rel. abundance (%)	Diff. Rel abundance (%)	95% lower CI	95% upper CI	p-values	Corrected p-values
**139**	Unclassified Leclercia	63	0.000122086	429	0.001081438	-0.000959352	-0.00106603	-0.000853	5.37E-91	1.06E-90
**140**	Unclassified Microbacteriaceae	0	0	27	6.81E-05	-6.81E-05	-9.37E-05	-4.24E-05	1.69E-10	2.70E-10
**141**	Unclassified Microbacterium	0	0	20	5.04E-05	-5.04E-05	-7.25E-05	-2.83E-05	5.79E-08	8.88E-08
**142**	Unclassified Micrococcales	8081	0.015659889	275	0.000693229	0.014966659	0.01461556	0.0153178	<1E-301	<1E-301
**143**	Unclassified Moraxellaceae	2015	0.003904798	2532	0.006382753	-0.002477954	-0.0027794	-0.002177	2.49E-61	4.69E-61
**144**	Unclassified Morganella	7	1.36E-05	3	7.56E-06	6.00E-06	-7.20E-06	1.92E-05	0.52949883	0.6830222
**145**	Unclassified Oceanospirillales	324	0.000627868	0	0	0.000627868	0.000559502	0.0006962	7.13E-81	1.39E-80
**146**	Unclassified Pantoea	92809	0.17985133	39616	0.099865375	0.079985955	0.078468541	0.0815034	<1E-301	<1E-301
**147**	Unclassified Pasteurellaceae	9735	0.018865118	7012	0.017676091	0.001189027	0.000630861	0.0017472	3.25E-05	4.76E-05
**148**	Unclassified Pectobacterium	4858	0.009414149	3284	0.00827842	0.001135729	0.000748129	0.0015233	1.18E-08	1.82E-08
**149**	Unclassified Photobacterium	1006	0.001949492	400	0.001008334	0.000941159	0.00078535	0.001097	5.21E-31	9.32E-31
**150**	Unclassified Photorhabdus	514	0.000996063	494	0.001245292	-0.000249229	-0.00038878	-0.00011	0.00041683	0.0005978
**151**	Unclassified Pluralibacter	8	1.55E-05	0	0	1.55E-05	4.76E-06	2.62E-05	0.01171299	0.0164738
**152**	Unclassified Proteobacteria	11275	0.02184943	8876	0.022374926	-0.000525496	-0.00114132	9.03E-05	0.09492099	0.1285266
**153**	Unclassified Providencia	6632	0.012851922	3318	0.008364128	0.004487793	0.004067497	0.0049081	2.12E-94	4.28E-94
**154**	Unclassified Pseudomonadaceae	25	4.84E-05	19	4.79E-05	5.51E-07	-2.82E-05	2.93E-05	1.00000036	1.0000004
**155**	Unclassified Pseudomonadales	497	0.000963119	386	0.000973042	-9.92E-06	-0.00013873	0.0001189	0.89201761	1.137192
**156**	Unclassified Pseudomonas	19005	0.036829128	11030	0.027804803	0.009024325	0.008287275	0.0097614	1.37E-124	2.93E-124
**157**	Unclassified Rahnella	431	0.00083522	338	0.000852042	-1.68E-05	-0.00013711	0.0001035	0.79905416	1.0246695
**158**	Unclassified Raoultella	5564	0.010782282	2553	0.00643569	0.004346592	0.003969002	0.0047242	5.32E-109	1.10E-108
**159**	Unclassified Rhizobiales	3727	0.007222424	2213	0.005578606	0.001643817	0.001315519	0.0019721	2.72E-22	4.49E-22
**160**	Unclassified Salmonella	2255	0.004369886	3110	0.007839795	-0.003469909	-0.00379921	-0.003141	1.01E-100	2.06E-100
**161**	Unclassified Serratia	11439	0.02216724	14893	0.037542786	-0.015375547	-0.01610246	-0.014649	<1E-301	<1E-301
**162**	Unclassified Shigella	6568	0.012727899	1239	0.003123314	0.009604585	0.009251057	0.0099581	<1E-301	<1E-301
**163**	Unclassified Staphylococcaceae	790	0.001530913	37	9.33E-05	0.001437643	0.00132674	0.0015485	3.14E-145	6.84E-145
**164**	Unclassified Staphylococcus	7841	0.015194801	4794	0.01208488	0.003109921	0.002630225	0.0035896	2.53E-36	4.55E-36
**165**	Unclassified Stenotrophomonas	0	0	1320	0.003327501	-0.003327501	-0.003507	-0.003148	<1E-301	<1E-301
**166**	Unclassified Vibrio	8801	0.017055151	6523	0.016443403	0.000611749	7.68E-05	0.0011467	0.02556166	0.0352686
**167**	Unclassified Vibrionaceae	225	0.00043602	160	0.000403334	3.27E-05	-5.19E-05	0.0001173	0.47187469	0.6123136
**168**	Unclassified Vibrionales	9	1.74E-05	16	4.03E-05	-2.29E-05	-4.57E-05	-8.01E-08	0.04402571	0.059985

S/N	Bacterial species	FEN_Res no. Reads	FEN_Res Rel. abundance (%)	FEN_Sus no. reads	FEN_Sus Rel. abundance (%)	Diff. Rel abundance (%)	95% lower CI	95% upper CI	p-values	Corrected p-values
169	Unclassified Xanthomonadaceae	0	0	1171	0.002951897	-0.002951897	-0.00312097	-0.002783	<1E-301	<1E-301
170	Unclassified Xanthomonadales	0	0	17	4.29E-05	-4.29E-05	-6.32E-05	-2.25E-05	7.05E-07	1.05E-06
171	Unclassified Xenorhabdus	1518	0.002941679	1241	0.003128355	-0.000186676	-0.00041513	4.18E-05	0.11095129	0.1493048
172	Unclassified Yersinia	7245	0.014039833	6455	0.016271986	-0.002232153	-0.00274406	-0.00172	7.66E-18	1.26E-17
**173**	**Unclassified Bacteria**	**90033**	**0.174471816**	**68339**	**0.172271301**	**0.002200515**	**0.000479501**	**0.0039215**	**0.01228673**	**0.0170606**
	**Total**	**51603177**	**100**	**39669405**	**100**					

S/N	Bacterial genera	FEN_Res no. reads	FEN_Res Rel. abundance (%)	FEN_Sus no. reads	FEN_Sus Rel. abundance (%)	Diff. Rel abundance (%)	95% lower CI	95% upper CI	p-values	Corrected p-values
**1**	*Acinetobacter*	3994881	7.741540797	4725882	11.91316583	-4.171625029	-4.18406642	-4.159184	<1E-301	<1E-301
**2**	*Bacillus*	235239	0.455861468	0	0	0.455861468	0.454023516	0.4576994	<1E-301	<1E-301
**3**	*Bradyrhizobium*	13940	0.027013841	17281	0.043562539	-0.016548698	-0.01733781	-0.01576	<1E-301	<1E-301
**4**	*Cedecea*	6042	0.011708581	5632	0.014197339	-0.002488758	-0.00296269	-0.002015	3.05E-25	6.66E-25
**5**	*Citrobacter*	117011	0.226751543	109514	0.276066656	-0.049315114	-0.05140081	-0.047229	<1E-301	<1E-301
**6**	*Cronobacter*	26967	0.05225841	21986	0.055423065	-0.003164654	-0.00412654	-0.002203	1.03E-10	2.02E-10
**7**	*Edwardsiella*	18006	0.0348932	9424	0.023756343	0.011136857	0.010437103	0.0118366	7.82E-208	2.94E-207
**8**	*Enterobacter*	2870703	5.563035392	10746972	27.09133651	-21.52830112	-21.5434794	-21.51312	<1E-301	<1E-301
**9**	*Erwinia*	16116	0.031230635	10257	0.025856198	0.005374437	0.004679647	0.0060692	3.80E-51	9.65E-51
**10**	*Escherichia*	1155297	2.238809831	732350	1.846133059	0.392676772	0.386859521	0.398494	<1E-301	<1E-301
**11**	*Haemophilus*	8778	0.017010581	4674	0.01178238	0.005228201	0.004737596	0.0057188	2.40E-94	7.53E-94
**12**	*Halomonas*	5818	0.0112745	0	0	0.0112745	0.010984809	0.0115642	<1E-301	<1E-301
**13**	*Klebsiella*	40029326	77.57143712	20796121	52.42357681	25.14786031	25.12859794	25.167123	<1E-301	<1E-301
**14**	*Kluyvera*	18385	0.035627651	15422	0.038876308	-0.003248656	-0.00404956	-0.002448	1.47E-15	2.95E-15
**15**	*Kosakonia*	25167	0.048770253	18218	0.045924561	0.002845692	0.001947141	0.0037442	5.99E-10	1.10E-09
**16**	*Leclercia*	6741	0.013063149	16607	0.041863497	-0.028800347	-0.02950919	-0.028092	<1E-301	<1E-301
**17**	*Lelliottia*	0	0	15489	0.039045204	-0.039045204	-0.03965998	-0.03843	<1E-301	<1E-301
**18**	*Microbacterium*	0	0	6685	0.016851778	-0.016851778	-0.01725571	-0.016448	<1E-301	<1E-301
**19**	*Morganella*	7529	0.014590187	6012	0.015155256	-0.000565069	-0.00107037	-5.98E-05	0.02829376	0.042897

S/N	Bacterial species	FEN_Res no. Reads	FEN_Res Rel. abundance (%)	FEN_Sus no. reads	FEN_Sus Rel. abundance (%)	Diff. Rel abundance (%)	95% lower CI	95% upper CI	p-values	Corrected p-values
**20**	*Pantoea*	101023	0.195768954	39616	0.099865375	0.095903579	0.094347753	0.0974594	<1E-301	<1E-301
**21**	*Pectobacterium*	10816	0.02095995	8859	0.022332072	-0.001372122	-0.00198221	-0.000762	1.01E-05	1.66E-05
**22**	*Photobacterium*	8122	0.015739341	4879	0.012299151	0.00344019	0.00295415	0.0039262	6.02E-43	1.49E-42
**23**	*Photorhabdus*	11705	0.022682712	7046	0.017761799	0.004920913	0.004337144	0.0055047	3.75E-60	9.78E-60
**24**	*Pluralibacter*	22702	0.043993415	12853	0.032400284	0.011593131	0.010792503	0.0123938	3.40E-173	1.23E-172
**25**	*Providencia*	14106	0.027335526	8538	0.021522884	0.005812642	0.005170916	0.0064544	3.26E-69	9.28E-69
**26**	*Pseudomonas*	19005	0.036829128	11030	0.027804803	0.009024325	0.008287275	0.0097614	1.37E-124	4.44E-124
**27**	*Rahnella*	33450	0.06482159	18377	0.046325373	0.018496217	0.017531527	0.0194609	1.54E-301	6.04E-301
**28**	*Raoultella*	53266	0.103222327	30026	0.075690573	0.027531754	0.026306998	0.0287565	<1E-301	<1E-301
**29**	*Salmonella*	380596	0.737543737	284694	0.717666423	0.019877314	0.016363096	0.0233915	1.70E-28	3.82E-28
**30**	*Serratia*	26886	0.052101443	45503	0.114705527	-0.062604084	-0.06382766	-0.061381	<1E-301	<1E-301
**31**	*Shigella*	50309	0.097492059	37410	0.094304414	0.003187645	0.001908037	0.0044673	1.10E-06	1.85E-06
**32**	*Staphylococcus*	7841	0.015194801	4794	0.01208488	0.003109921	0.002630225	0.0035896	2.53E-36	5.94E-36
**33**	*Stenotrophomonas*	0	0	6179	0.015576236	-0.015576236	-0.01596458	-0.015188	<1E-301	<1E-301
**34**	*Vibrio*	8801	0.017055151	6523	0.016443403	0.000611749	7.68E-05	0.0011467	0.02556166	0.0393901
**35**	*Xenorhabdus*	8675	0.01681098	7370	0.018578549	-0.001767569	-0.00231984	-0.001215	2.97E-10	5.58E-10
**36**	*Yersinia*	7245	0.014039833	6455	0.016271986	-0.002232153	-0.00274406	-0.00172	7.66E-18	1.56E-17
**37**	*Yokenella*	13422	0.026010026	11025	0.027792199	-0.001782173	-0.00246234	-0.001102	2.62E-07	4.56E-07
**38**	*Uncultured beta proteobacterium CBNPD1 BAC clone 578*	8263	0.01601258	5320	0.013410839	0.002601741	0.002102711	0.0031008	3.61E-24	7.72E-24
**39**	Unclassified Actinobacteria	11020	0.021355274	1805	0.004550106	0.016805168	0.016354614	0.0172557	<1E-301	<1E-301
**40**	Unclassified Actinobacteria	413	0.000800338	31	7.81E-05	0.000722192	0.00064025	0.0008041	3.44E-66	9.50E-66
**41**	Unclassified Alphaproteobacteria	3094	0.005995755	2473	0.006234023	-0.000238269	-0.0005623	8.58E-05	0.15184486	0.219591
**42**	Unclassified Bacillaceae	558	0.001081329	0	0	0.001081329	0.000991609	0.001171	9.72E-139	3.26E-138
**43**	Unclassified Bacillales	1110	0.00215103	1361	0.003430856	-0.001279825	-0.00150171	-0.001058	5.36E-31	1.23E-30
**44**	Unclassified Bacilli	2698	0.00522836	134	0.000337792	0.004890568	0.004685166	0.005096	<1E-301	<1E-301
**45**	Unclassified Bacteroidetes	5888	0.01141015	386	0.000973042	0.010437108	0.010129939	0.0107443	<1E-301	<1E-301
**46**	Unclassified Betaproteobacteria	738	0.001430145	602	0.001517542	-8.74E-05	-0.00024659	7.18E-05	0.28262625	0.3965204
**47**	Unclassified Bradyrhizobiaceae	281	0.00054454	329	0.000829355	-0.000284814	-0.00039475	-0.000175	2.42E-07	4.29E-07

S/N	Bacterial species	FEN_Res no. Reads	FEN_Res Rel. abundance (%)	FEN_Sus no. reads	FEN_Sus Rel. abundance (%)	Diff. Rel abundance (%)	95% lower CI	95% upper CI	p-values	Corrected p-values
**48**	Unclassified Burkholderiales	5567	0.010788095	4399	0.01108915	-0.000301055	-0.00073427	0.0001322	0.17255564	0.2457611
**49**	Unclassified Enterobacteriaceae	2016336	3.90738733	1692643	4.266872669	-0.359485339	-0.3677016	-0.351269	<1E-301	<1E-301
**50**	Unclassified Enterobacteriales	1	1.94E-06	0	0	1.94E-06	-1.86E-06	5.74E-06	0.99999995	1.3239436
**51**	Unclassified Firmicutes	4159	0.008059581	2258	0.005692044	0.002367537	0.00202826	0.0027068	1.72E-41	4.15E-41
**52**	Unclassified Flavobacteriaceae	0	0	4210	0.010612713	-0.010612713	-0.01093327	-0.010292	<1E-301	<1E-301
**53**	Unclassified Flavobacteriales	0	0	1	2.52E-06	-2.52E-06	-7.46E-06	2.42E-06	0.4346256	0.600806
**54**	Unclassified Gammaproteobacteria	70864	0.137324878	48595	0.122499947	0.014824932	0.013339781	0.0163101	1.92E-84	5.81E-84
**55**	Unclassified Halomonadaceae	8	1.55E-05	0	0	1.55E-05	4.76E-06	2.62E-05	0.01171299	0.0186614
**56**	Unclassified Intrasporangiaceae	5570	0.010793909	0	0	0.010793909	0.010510459	0.0110774	<1E-301	<1E-301
**57**	Unclassified Lactobacillales	5957	0.011543863	4075	0.0102724	0.001271463	0.000840894	0.001702	8.82E-09	1.59E-08
**58**	Unclassified Microbacteriaceae	0	0	27	6.81E-05	-6.81E-05	-9.37E-05	-4.24E-05	1.69E-10	3.25E-10
**59**	Unclassified Micrococcales	8081	0.015659889	275	0.000693229	0.014966659	0.01461556	0.0153178	<1E-301	<1E-301
**60**	Unclassified Moraxellaceae	2015	0.003904798	2532	0.006382753	-0.002477954	-0.0027794	-0.002177	2.49E-61	6.70E-61
**61**	Unclassified Oceanospirillales	324	0.000627868	0	0	0.000627868	0.000559502	0.0006962	7.13E-81	2.09E-80
**62**	Unclassified Pasteurellaceae	9735	0.018865118	7012	0.017676091	0.001189027	0.000630861	0.0017472	3.25E-05	5.27E-05
**63**	Unclassified Proteobacteria	11275	0.02184943	8876	0.022374926	-0.000525496	-0.00114132	9.03E-05	0.09492099	0.1394152
**64**	Unclassified Pseudomonadaceae	25	4.84E-05	19	4.79E-05	5.51E-07	-2.82E-05	2.93E-05	1.00000036	1.0000004
**65**	Unclassified Pseudomonadales	497	0.000963119	386	0.000973042	-9.92E-06	-0.00013873	0.0001189	0.89201761	1.1978522
**66**	Unclassified Rhizobiales	3727	0.007222424	2213	0.005578606	0.001643817	0.001315519	0.0019721	2.72E-22	5.68E-22
**67**	Unclassified Staphylococcaceae	790	0.001530913	37	9.33E-05	0.001437643	0.00132674	0.0015485	3.14E-145	1.09E-144
**68**	Unclassified Vibrionaceae	225	0.00043602	160	0.000403334	3.27E-05	-5.19E-05	0.0001173	0.47187469	0.6428438
**69**	Unclassified Vibrionales	9	1.74E-05	16	4.03E-05	-2.29E-05	-4.57E-05	-8.01E-08	0.04402571	0.0656892
**70**	Unclassified Xanthomonadaceae	0	0	1171	0.002951897	-0.002951897	-0.00312097	-0.002783	<1E-301	<1E-301
**71**	Unclassified Xanthomonadales	0	0	17	4.29E-05	-4.29E-05	-6.32E-05	-2.25E-05	7.05E-07	1.20E-06
**72**	**Unclassified Bacteria**	**90033**	**0.174471816**	**68339**	**0.172271301**	**0.002200515**	**0.000479501**	**0.0039215**	**0.01228673**	**0.0192492**
	**Total**	**51603177**	**100**	**39669405**	**100**					

S/N	Bacterial species	FEN_Res no. Reads	FEN_Res Rel. abundance (%)	FEN_Sus no. reads	FEN_Sus Rel. abundance (%)	Diff. Rel abundance (%)	95% lower CI	95% upper CI	p-values	Corrected p-values
S/N	Bacterial family	FE_Res no. Reads	FEN_Res Rel abundance (%)	FEN_Sus no. Reads	FEN_Res Re. abundance (%)	Diff. Rel abundance (%)	95% lower CI	95% upper CI	p-values	Corrected p-values
1	Bacillaceae	235797	0.456942796	0	0	0.456942796	0.455102676	0.4587829	<1E-301	<1E-301
2	Bradyrhizobiaceae	14221	0.027558381	17610	0.044391893	-0.016833513	-0.01763024	-0.016037	<1E-301	<1E-301
3	Enterobacteriaceae	47047827	91.17234584	34714419	87.50930093	3.663044909	3.650170062	3.6759198	<1E-301	<1E-301
4	Flavobacteriaceae	0	0	4210	0.010612713	-0.010612713	-0.01093327	-0.010292	<1E-301	<1E-301
5	Halomonadaceae	5826	0.011290003	0	0	0.011290003	0.011000113	0.0115799	<1E-301	<1E-301
6	Intrasporangiaceae	5570	0.010793909	0	0	0.010793909	0.010510459	0.0110774	<1E-301	<1E-301
7	Microbacteriaceae	0	0	6712	0.01691984	-0.01691984	-0.01732459	-0.016515	<1E-301	<1E-301
8	Moraxellaceae	3996896	7.745445595	4728414	11.91954858	-4.174102984	-4.18654726	-4.161659	<1E-301	<1E-301
9	Pasteurellaceae	18513	0.035875698	11686	0.029458471	0.006417228	0.005674157	0.0071603	2.87E-63	7.41E-63
10	Pseudomonadaceae	19030	0.036877574	11049	0.027852699	0.009024875	0.008287267	0.0097625	2.07E-124	7.24E-124
11	Staphylococcaceae	8631	0.016725714	4831	0.012178151	0.004547563	0.004055218	0.0050399	1.55E-71	4.48E-71
12	Vibrionaceae	17148	0.033230512	11562	0.029145887	0.004084625	0.003356991	0.0048123	7.35E-28	1.64E-27
13	Xanthomonadaceae	0	0	7350	0.018528133	-0.018528133	-0.01895167	-0.018105	<1E-301	<1E-301
14	Uncultured beta proteobacterium CBNPD1 BAC clone 578	8263	0.01601258	5320	0.013410839	0.002601741	0.002102711	0.0031008	3.61E-24	7.70E-24
15	Unclassified Actinobacteria	11020	0.021355274	1805	0.004550106	0.016805168	0.016354614	0.0172557	<1E-301	<1E-301
16	Unclassified Actinobacteria	413	0.000800338	31	7.81E-05	0.000722192	0.00064025	0.0008041	3.44E-66	9.36E-66
17	Unclassified Alphaproteobacteria	3094	0.005995755	2473	0.006234023	-0.000238269	-0.0005623	8.58E-05	0.15184486	0.2480133
18	Unclassified Bacillales	1110	0.00215103	1361	0.003430856	-0.001279825	-0.00150171	-0.001058	5.36E-31	1.25E-30
19	Unclassified Bacilli	2698	0.00522836	134	0.000337792	0.004890568	0.004685166	0.005096	<1E-301	<1E-301
20	Unclassified Bacteroidetes	5888	0.01141015	386	0.000973042	0.010437108	0.010129939	0.0107443	<1E-301	<1E-301
21	Unclassified Betaproteobacteria	738	0.001430145	602	0.001517542	-8.74E-05	-0.00024659	7.18E-05	0.28262625	0.4327714
22	Unclassified Burkholderiales	5567	0.010788095	4399	0.01108915	-0.000301055	-0.00073427	0.0001322	0.17255564	0.2727492
23	Unclassified Enterobacteriales	1	1.94E-06	0	0	1.94E-06	-1.86E-06	5.74E-06	0.99999995	1.3999999
24	Unclassified Firmicutes	4159	0.008059581	2258	0.005692044	0.002367537	0.00202826	0.0027068	1.72E-41	4.21E-41
25	Unclassified Flavobacteriales	0	0	1	2.52E-06	-2.52E-06	-7.46E-06	2.42E-06	0.4346256	0.6453532

S/N	Bacterial species	FEN_Res no. Reads	FEN_Res Rel. abundance (%)	FEN_Sus no. reads	FEN_Sus Rel. abundance (%)	Diff. Rel abundance (%)	95% lower CI	95% upper CI	p-values	Corrected p-values
26	Unclassified Gammaproteobacteria	70864	0.137324878	48595	0.122499947	0.014824932	0.013339781	0.0163101	1.92E-84	6.26E-84
27	Unclassified Lactobacillales	5957	0.011543863	4075	0.0102724	0.001271463	0.000840894	0.001702	8.82E-09	1.73E-08
28	Unclassified Micrococcales	8081	0.015659889	275	0.000693229	0.014966659	0.01461556	0.0153178	<1E-301	<1E-301
29	Unclassified Oceanospirillales	324	0.000627868	0	0	0.000627868	0.000559502	0.0006962	7.13E-81	2.18E-80
30	Unclassified Proteobacteria	11275	0.02184943	8876	0.022374926	-0.000525496	-0.00114132	9.03E-05	0.09492099	0.1603837
31	Unclassified Pseudomonadales	497	0.000963119	386	0.000973042	-9.92E-06	-0.00013873	0.0001189	0.89201761	1.2855548
32	Unclassified Rhizobiales	3727	0.007222424	2213	0.005578606	0.001643817	0.001315519	0.0019721	2.72E-22	5.55E-22
33	Unclassified Vibrionales	9	1.74E-05	16	4.03E-05	-2.29E-05	-4.57E-05	-8.01E-08	0.04402571	0.077045
34	Unclassified Xanthomonadales	0	0	17	4.29E-05	-4.29E-05	-6.32E-05	-2.25E-05	7.05E-07	1.33E-06
**35**	**Unclassified Bacteria**	**90033**	**0.174471816**	**68339**	**0.172271301**	**0.002200515**	**0.000479501**	**0.0039215**	**0.01228673**	**0.0222981**
	**Total**	**51603177**	**100**	**39669405**	**100**					

S/N	Bacterial order	FEN_Res no. Reads	FEN_Res Rel. abundance (%)	FEN_Sus no. Reads	FEN_SusL Rel. abundance (%)	Diff. Rel abundance (%)	95% lower CI	95% upper CI	p-values	Corrected p-values
1	Bacillales	245538	0.475819541	6192	0.015609006	0.460210535	0.458293145	0.4621279	<1E-301	<1E-301
2	Burkholderiales	5567	0.010788095	4399	0.01108915	-0.000301055	-0.00073427	0.0001322	0.17255564	0.2353031
3	Enterobacteriales	47047828	91.17234778	34714419	87.50930093	3.663046847	3.650172	3.6759217	<1E-301	<1E-301
4	Flavobacteriales	0	0	4211	0.010615234	-0.010615234	-0.01093583	-0.010295	<1E-301	<1E-301
5	Lactobacillales	5957	0.011543863	4075	0.0102724	0.001271463	0.000840894	0.001702	8.82E-09	1.47E-08
6	Micrococcales	13651	0.026453798	6987	0.01761307	0.008840728	0.008234588	0.0094469	1.05E-174	2.87E-174
7	Oceanospirillales	6150	0.011917871	0	0	0.011917871	0.011620031	0.0122157	<1E-301	<1E-301
8	Pasteurellales	18513	0.035875698	11686	0.029458471	0.006417228	0.005674157	0.0071603	2.87E-63	6.16E-63
9	Pseudomonadales	4016423	7.783286289	4739849	11.94837432	-4.165088031	-4.17755039	-4.152626	<1E-301	<1E-301
10	Rhizobiales	17948	0.034780804	19823	0.0499705	-0.015189696	-0.01605137	-0.014328	3.56E-271	1.07E-270
11	Vibrionales	17157	0.033247953	11578	0.02918622	0.004061732	0.003333742	0.0047897	1.59E-27	2.98E-27
12	Xanthomonadales	0	0	7367	0.018570987	-0.018570987	-0.01899502	-0.018147	<1E-301	<1E-301

S/N	Bacterial species	FEN_Res no. Reads	FEN_Res Rel. abundance (%)	FEN_Sus no. reads	FEN_Sus Rel. abundance (%)	Diff. Rel abundance (%)	95% lower CI	95% upper CI	p-values	Corrected p-values
13	Uncultured beta proteobacterium CBNPD1 BAC clone 578	8263	0.01601258	5320	0.013410839	0.002601741	0.002102711	0.0031008	3.61E-24	6.38E-24
14	Unclassified Actinobacteria	11020	0.021355274	1805	0.004550106	0.016805168	0.016354614	0.0172557	<1E-301	<1E-301
15	Unclassified Actinobacteria	413	0.000800338	31	7.81E-05	0.000722192	0.00064025	0.0008041	3.44E-66	7.93E-66
16	Unclassified Alphaproteobacteria	3094	0.005995755	2473	0.006234023	-0.000238269	-0.0005623	8.58E-05	0.15184486	0.2169212
17	Unclassified Bacilli	2698	0.00522836	134	0.000337792	0.004890568	0.004685166	0.005096	<1E-301	<1E-301
18	Unclassified Bacteroidetes	5888	0.01141015	386	0.000973042	0.010437108	0.010129939	0.0107443	<1E-301	<1E-301
19	Unclassified Betaproteobacteria	738	0.001430145	602	0.001517542	-8.74E-05	-0.00024659	7.18E-05	0.28262625	0.3686429
20	Unclassified Firmicutes	4159	0.008059581	2258	0.005692044	0.002367537	0.00202826	0.0027068	1.72E-41	3.44E-41
21	Unclassified Gammaproteobacteria	70864	0.137324878	48595	0.122499947	0.014824932	0.013339781	0.0163101	1.92E-84	4.79E-84
22	Unclassified Proteobacteria	11275	0.02184943	8876	0.022374926	-0.000525496	-0.00114132	9.03E-05	0.09492099	0.1423815
**23**	**Unclassified Bacteria**	**90033**	**0.174471816**	**68339**	**0.172271301**	**0.002200515**	**0.000479501**	**0.0039215**	**0.01228673**	**0.0194001**
	**Total**	**51603177**	**100**	**39669405**	**100**					

S/N	Bacterial Class	FEN_Res no. reads	FEN_Res Rel. abundance (%)	FEN_Sus no. reads	FEN_Sus Rel. abundance (%)	Diff. Rel abundance (%)	95% lower CI	95% upper CI	p-values	Corrected p-values
1	Actinobacteria	24671	0.047809072	8792	0.022163176	0.025645896	0.024890711	0.0264011	<1E-301	<1E-301
2	Alphaproteobacteria	21042	0.040776559	22296	0.056204523	-0.015427964	-0.0163485	-0.014507	3.55E-244	7.70E-244
3	Bacilli	254193	0.492591764	10401	0.026219198	0.466372566	0.464397032	0.4683481	<1E-301	<1E-301
4	Betaproteobacteria	14568	0.02823082	10321	0.026017532	0.002213288	0.001533599	0.002893	2.06E-10	2.97E-10
5	Flavobacteriia	0	0	4211	0.010615234	-0.010615234	-0.01093583	-0.010295	<1E-301	<1E-301
6	Gammaproteobacteria	51176935	99.17400047	39533494	99.65739088	-0.483390409	-0.48645708	-0.480324	<1E-301	<1E-301
7	Unclassified Actinobacteria	413	0.000800338	31	7.81E-05	0.000722192	0.00064025	0.0008041	3.44E-66	6.38E-66
8	Unclassified Bacteroidetes	5888	0.01141015	386	0.000973042	0.010437108	0.010129939	0.0107443	<1E-301	<1E-301
9	Unclassified Firmicutes	4159	0.008059581	2258	0.005692044	0.002367537	0.00202826	0.0027068	1.72E-41	2.80E-41
10	Unclassified Proteobacteria	11275	0.02184943	8876	0.022374926	-0.000525496	-0.00114132	9.03E-05	0.09492099	0.1121793

S/N	Bacterial species	FEN_Res no. Reads	FEN_Res Rel. abundance (%)	FEN_Sus no. reads	FEN_Sus Rel. abundance (%)	Diff. Rel abundance (%)	95% lower CI	95% upper CI	p-values	Corrected p-values
**11**	**Unclassified Bacteria**	**90033**	**0.174471816**	**68339**	**0.172271301**	**0.002200515**	**0.000479501**	**0.0039215**	**0.01228673**	**0.0159728**
	**Total**	**51603177**	**100**	**39669405**	**100**					

S/N	Bacterial Phylum	FEN_Res no. reads	FEN_Res Rel. abundance (%)	FEN_Sus no. reads	FEN_Sus Rel. abundance (%)	Diff. Rel abundance (%)	95% lower CI	95% upper CI	p-values	Corrected p-values
**1**	Firmicutes	258352	0.500651346	12659	0.031911242	0.468740103	0.466735802	0.4707444	<1E-301	<1E-301
**2**	Actinobacteria	25084	0.04860941	8823	0.022241322	0.026368088	0.025608474	0.0271277	<1E-301	<1E-301
**3**	Bacteroidetes	5888	0.01141015	4597	0.011588276	-0.000178125	-0.00062212	0.0002659	0.43066824	0.4306682
**4**	Proteobacteria	51223820	99.26485728	39574987	99.76198786	-0.497130581	-0.49991117	-0.49435	<1E-301	<1E-301
**5**	**Unclassified Bacteria**	**90033**	**0.174471816**	**68339**	**0.172271301**	**0.002200515**	**0.000479501**	**0.0039215**	**0.01228673**	**0.0153584**
	**Total**	**51603177**	**100**	**39669405**	**100**					

Supplementary Table 2: Relative abundances of all enzyme classes detected in FEN_Res and FEN_Sus.

S/N	Bacterial xenobiotic degradation enzymes	FEN_Res Rel. abundance (%)	FEN_Sus Rel. abundance (%)	Diff. bw Rel. abundance	95% lower CI	95% upper CI	p-values	Corrected p-values
**Oxidoreductases**								
1	K00244 fumarate reductase flavoprotein subunit	4.91088366	5.385051888	-0.47417	-0.575095543	-0.373240909	2.12E-20	1.06E-19
2	K04072 acetaldehyde dehydrogenase / alcohol dehydrogenase	2.64296336	3.140879523	-0.49792	-0.574740468	-0.421091852	9.23E-38	1.00E-36

S/N	Bacterial species	FEN_Res no. Reads	FEN_Res Rel. abundance (%)	FEN_Sus no. reads	FEN_Sus Rel. abundance (%)	Diff. Rel abundance (%)	95% lower CI	95% upper CI	p-values	Corrected p-values
3	K00121 S-(hydroxymethyl)glutathione dehydrogenase / alcohol dehydrogenase	2.6377302	2.795918171	-0.15819	-0.232337847	-0.084038086	2.74E-05	5.31E-05		
4	K00074 3-hydroxybutyryl-CoA dehydrogenase	2.5487665	2.462498277	0.086268	0.015285701	0.157250747	0.017686561	0.0252665		
5	K00239 succinate dehydrogenase / fumarate reductase, flavoprotein subunit	2.42447897	2.751034724	-0.32656	-0.399194669	-0.253916831	6.88E-19	3.20E-18		
41	K03863 vanillate O-demethylase ferredoxin subunit	2.32897382	2.137734476	0.191239	0.124292878	0.258185812	2.66E-08	6.92E-08		
6	K00088 IMP dehydrogenase	1.82026715	2.494878478	-0.67461	-0.741640212	-0.607582438	3.06E-90	1.33E-88		
7	K00146 phenylacetaldehyde dehydrogenase	1.72367176	1.546555356	0.177116	0.119722018	0.234510788	1.96E-09	5.80E-09		
8	K00481 p-hydroxybenzoate 3-monooxygenase	1.53898485	1.05732578	0.481659	0.431085964	0.532232185	1.45E-74	3.77E-73		
9	K05549 benzoate/toluate 1,2-dioxygenase alpha subunit	1.45329188	1.615162911	-0.16187	-0.218055167	-0.105686905	1.31E-08	3.48E-08		
10	K00245 fumarate reductase iron-sulfur subunit	1.37501254	1.259301293	0.115711	0.064065563	0.167356927	1.30E-05	2.61E-05		
11	K10680 N-ethylmaleimide reductase	1.28822932	1.449735348	-0.16151	-0.214653915	-0.108358147	2.01E-09	5.81E-09		
12	K03862 vanillate O-demethylase monooxygenase subunit	1.19795732	1.334641365	-0.13668	-0.187803172	-0.085564911	1.37E-07	3.30E-07		
13	K00449 protocatechuate 3,4-dioxygenase, beta subunit	0.9652998	0.906966232	0.058334	0.014657455	0.102009671	0.009447966	0.0144498		
14	K00055 aryl-alcohol dehydrogenase	0.94218668	0.92331663	0.01887	-0.024814957	0.062555048	0.405318079	0.4503534		

S/N	Bacterial species	FEN_Res no. Reads	FEN_Res Rel. abundance (%)	FEN_Sus no. reads	FEN_Sus Rel. abundance (%)	Diff. Rel abundance (%)	95% lower CI	95% upper CI	p-values	Corrected p-values
15	K05784 benzoate/toluate 1,2-dioxygenase reductase subunit	0.89879507	0.970764846	-0.07197	-0.1159019	-0.028037662	0.001283209	0.0020852		
16	K00001 alcohol dehydrogenase	0.88636631	0.831626159	0.05474	0.012888777	0.096591529	0.010922916	0.0163216		
17	K03381 catechol 1,2-dioxygenase	0.88505802	0.970764846	-0.08571	-0.129510132	-0.041903514	0.000116189	0.0002041		
18	K10679 nitroreductase / dihydropteridine reductase	0.87524585	1.029113328	-0.15387	-0.198376781	-0.109358176	7.75E-12	2.40E-11		
19	K10678 nitroreductase	0.87262927	0.790910461	0.081719	0.040598212	0.122839407	0.000110248	0.0001963		
20	K13953 alcohol dehydrogenase, propanol-preferring	0.80677869	0.889974641	-0.0832	-0.125108057	-0.041283854	9.02E-05	0.0001651		
21	K00247 fumarate reductase subunit D	0.74986808	0.662351444	0.087517	0.049652175	0.125381099	7.03E-06	1.43E-05		
22	K05783 dihydroxycyclohexadiene carboxylate dehydrogenase	0.74441687	0.670045749	0.074371	0.036442648	0.112299601	0.000142415	0.0002469		
23	K00448 protocatechuate 3,4-dioxygenase, alpha subunit	0.72980764	0.8120698	-0.08226	-0.122247547	-0.042276778	5.02E-05	9.46E-05		
24	K00240 succinate dehydrogenase / fumarate reductase, iron-sulfur subunit	0.64237027	1.037128229	-0.39476	-0.437168369	-0.352347544	2.29E-79	7.44E-78		
25	K00246 fumarate reductase subunit C	0.62361812	0.518724412	0.104894	0.070913943	0.13887347	2.33E-09	6.58E-09		
26	K05550 benzoate/toluate 1,2-dioxygenase beta subunit	0.51633836	0.582843623	-0.06651	-0.100326636	-0.032683892	0.000102585	0.0001852		
27	K00242 succinate dehydrogenase / fumarate reductase, membrane anchor subunit	0.49954864	0.629971243	-0.13042	-0.164879837	-0.095965368	5.32E-14	1.87E-13		

S/N	Bacterial species	FEN_Res no. Reads	FEN_Res Rel. abundance (%)	FEN_Sus no. reads	FEN_Sus Rel. abundance (%)	Diff. Rel abundance (%)	95% lower CI	95% upper CI	p-values	Corrected p-values
28	K00241 succinate dehydrogenase / fumarate reductase, cytochrome b subunit	0.41232932	0.579637662	-0.16731	-0.199768645	-0.134848034	4.98E-25	2.94E-24		
29	K00257 Unclassified oxidoreductase	0.33165145	0.548219249	-0.21657	-0.247362914	-0.185772676	2.89E-46	4.17E-45		
30	K06446 acyl-CoA dehydrogenase	0.27561304	0.432484074	-0.15687	-0.18444908	-0.129292983	1.23E-30	8.43E-30		
31	K13954 alcohol dehydrogenase	0.22044683	0.316107707	-0.09566	-0.119584057	-0.071737708	9.59E-16	4.15E-15		
32	K11731 citronellyl-CoA dehydrogenase	0.15786696	0.253270881	-0.0954	-0.116455087	-0.074352747	4.07E-20	1.96E-19		
33	K00252 glutaryl-CoA dehydrogenase	0.10837	0.176327829	-0.06796	-0.08549207	-0.050423584	4.13E-15	1.68E-14		
34	K00128 aldehyde dehydrogenase (NAD+)	0.10815195	0.150038952	-0.04189	-0.058470028	-0.02530397	4.10E-07	8.87E-07		
35	K05599 anthranilate 1,2-dioxygenase (deaminating, decarboxylating) large subunit	0.08416664	0.122788288	-0.03862	-0.053503705	-0.023739588	2.03E-07	4.54E-07		
36	K13774 citronellol/citronellal dehydrogenase	0.08373055	0.117979347	-0.03425	-0.048918593	-0.019579012	3.22E-06	6.64E-06		
37	K11311 anthranilate 1,2-dioxygenase reductase subunit	0.06781302	0.112208618	-0.0444	-0.058352657	-0.030438541	1.09E-10	3.30E-10		
38	K00100 Unclassified oxidoreductase	0.04208332	0.067645767	-0.02556	-0.036447683	-0.01467721	2.19E-06	4.58E-06		
39	K05600 anthranilate 1,2-dioxygenase (deaminating, decarboxylating) small subunit	0.03030871	0.055142521	-0.02483	-0.034490582	-0.015177034	1.63E-07	3.79E-07		
40	K00517 Unclassified oxidoreductase	0.02420336	0.003205961	0.020997	0.016076364	0.025918436	2.08E-15	8.71E-15		
**Transferases**										

S/N	Bacterial species	FEN_Res no. Reads	FEN_Res Rel. abundance (%)	FEN_Sus no. reads	FEN_Sus Rel. abundance (%)	Diff. Rel abundance (%)	95% lower CI	95% upper CI	p-values	Corrected p-values
1	K00799 glutathione S-transferase	5.20895568	4.631009974	0.577946	0.480093279	0.675798133	1.42E-30	9.22E-30		
2	K00626 acetyl-CoA C-acetyltransferase	4.66623348	5.052914378	-0.38668	-0.484837279	-0.288524523	8.69E-15	3.23E-14		
3	K00632 acetyl-CoA acyltransferase	3.50272778	3.021297196	0.481431	0.401182989	0.561678188	2.24E-31	1.71E-30		
4	K00757 uridine phosphorylase	1.3798096	1.347785803	0.032024	-0.020676455	0.084724049	0.238269439	0.2922172		
5	K00758 thymidine phosphorylase	1.37195986	1.514495751	-0.14254	-0.197036884	-0.088034894	2.57E-07	5.66E-07		
6	K00760 hypoxanthine phosphoribosyltransferase	1.2014461	1.336885538	-0.13544	-0.186612751	-0.084266132	1.78E-07	4.06E-07		
7	K01026 propionate CoA-transferase	1.19926561	1.264110234	-0.06484	-0.115140041	-0.0145492	0.011359599	0.0167812		
8	K00876 uridine kinase	1.19752123	1.530846149	-0.33332	-0.386687101	-0.279962743	1.52E-35	1.41E-34		
9	K01031 3-oxoadipate CoA-transferase, alpha subunit	0.89203557	1.016930678	-0.1249	-0.169394506	-0.080395713	2.86E-08	7.29E-08		
10	K01032 3-oxoadipate CoA-transferase, beta subunit	0.78737239	0.948002526	-0.16063	-0.203183392	-0.118076886	6.88E-14	2.35E-13		
11	K00857 thymidine kinase	0.77080072	0.931972724	-0.16117	-0.203335312	-0.119008701	3.25E-14	1.17E-13		
12	K00680 Unclassified transferase	0.13802457	0.06411921	0.073905	0.059963736	0.087846982	1.70E-23	9.60E-23		
**Hydorlases**										
1	K01061 carboxymethylenebutenolidase	4.85942427	3.55284545	1.306579	1.21661968	1.39653795	4.38E-172	2.85E-170		
2	K01053 gluconolactonase	2.54331529	1.555852641	0.987463	0.924514515	1.050410789	4.80E-197	6.24E-195		
3	K01077 alkaline phosphatase	1.90029088	1.474100648	0.42619	0.368309681	0.484070776	7.58E-46	9.86E-45		
4	K01093 4-phytase / acid phosphatase	1.67112212	1.315726198	0.355396	0.300848231	0.409943613	2.42E-36	2.42E-35		
5	K01489 cytidine deaminase	1.05360935	1.010839353	0.04277	-0.003116287	0.088656289	0.070211784	0.0931381		
6	K03788 acid phosphatase (class B)	1.01915772	0.929087359	0.09007	0.045589323	0.134551405	8.10E-05	0.0001504		

S/N	Bacterial species	FEN_Res no. Reads	FEN_Res Rel. abundance (%)	FEN_Sus no. reads	FEN_Sus Rel. abundance (%)	Diff. Rel abundance (%)	95% lower CI	95% upper CI	p-values	Corrected p-values
7	K09474 acid phosphatase (class A)	0.96006664	0.869135897	0.090931	0.047831543	0.134029934	4.10E-05	7.84E-05		
8	K01428 urease subunit alpha	0.9386979	1.295208051	-0.35651	-0.405021554	-0.307998742	4.88E-49	7.93E-48		
9	K01055 3-oxoadipate enol-lactonase	0.76992852	0.838358676	-0.06843	-0.109219552	-0.027640754	0.000963722	0.0015859		
10	K01512 acylphosphatase	0.59548989	0.520006797	0.075483	0.04182431	0.109141875	1.35E-05	2.66E-05		
11	K01429 urease subunit beta	0.45572093	0.41741606	0.038305	0.008440033	0.068169713	0.012800671	0.0186976		
12	K01555 fumarylacetoacetase	0.20801807	0.32155784	-0.11354	-0.137385557	-0.08969398	7.51E-22	4.07E-21		
13	K01430 urease subunit gamma	0.16985962	0.379906322	-0.21005	-0.234707102	-0.185386301	3.34E-70	7.25E-69		
14	K01426 amidase	0.1155656	0.162862795	-0.0473	-0.06452905	-0.030065349	4.10E-08	1.02E-07		
15	K01113 alkaline phosphatase D	0.0898359	0.14458882	-0.05475	-0.070658673	-0.038847172	3.16E-12	1.00E-11		
16	K01457 allophanate hydrolase	0.04491795	0.05931027	-0.01439	-0.024909358	-0.003875285	0.006288849	0.0097327		
**Isomerases**										
1	K01856 muconate cycloisomerase	1.41295294	1.362212626	0.05074	-0.002378315	0.103858945	0.062626064	0.0839318		
2	K01857 3-carboxy-cis,cis-muconate cycloisomerase	0.70625842	0.7146086	-0.00835	-0.046575724	0.029875368	0.668267342	0.7120882		
3	K01826 5-carboxymethyl-2-hydroxymuconate isomerase	0.49126281	0.522571565	-0.03131	-0.063707604	0.001090083	0.0573043	0.0775996		
4	K01821 4-oxalocrotonate tautomerase	0.33535828	0.336946451	-0.00159	-0.027923178	0.024746826	0.904249357	0.9480034		
5	K03464 muconolactone D-isomerase	0.23331167	0.365479499	-0.13217	-0.157533846	-0.106801805	6.59E-26	4.08E-25		
6	K01800 maleylacetoacetate isomerase	0.07173789	0.166389351	-0.09465	-0.110918705	-0.07838422	5.96E-34	5.17E-33		
**Lyases**										
1	K01607 4-carboxymuconolactone decarboxylase	2.59412055	2.127154806	0.466966	0.39855145	0.535380029	6.77E-40	8.00E-39		
2	K01692 enoyl-CoA hydratase	1.00934555	1.199029235	-0.18968	-0.237599076	-0.141768295	4.44E-15	1.75E-14		

S/N	Bacterial species	FEN_Res no. Reads	FEN_Res Rel. abundance (%)	FEN_Sus no. reads	FEN_Sus Rel. abundance (%)	Diff. Rel abundance (%)	95% lower CI	95% upper CI	p-values	Corrected p-values
3	K01666 4-hydroxy 2-oxovalerate aldolase	0.86630587	0.888371661	-0.02207	-0.064535926	0.020404344	0.307002888	0.3628216		
4	K02554 2-keto-4-pentenoate hydratase	0.56191045	0.543410308	0.0185	-0.015169256	0.052169543	0.287918577	0.3433891		
5	K10218 4-hydroxy-4-methyl-2-oxoglutarate aldolase	0.415382	0.255515054	0.159867	0.134169364	0.185564526	1.71E-32	1.39E-31		
6	K01640 hydroxymethylglutaryl-CoA lyase	0.03314334	0.037189142	-0.00405	-0.012621111	0.004529508	0.351215401	0.4113334		
7	K01576 benzoylformate decarboxylase	0.00872193	0.052257157	-0.04354	-0.051998621	-0.035071829	5.41E-31	3.91E-30		
**Ligases**										
1	K01951 GMP synthase (glutamine-hydrolysing)	2.29495829	2.585286565	-0.29033	-0.360896031	-0.219760524	4.48E-16	2.01E-15		
2	K13778 geranyl-CoA carboxylase beta subunit	0.15372405	0.210952202	-0.05723	-0.076921211	-0.0375351	6.22E-09	1.68E-08		
3	K13776 citronellyl-CoA synthetase	0.13126507	0.201013725	-0.06975	-0.088639616	-0.050857688	9.83E-14	3.28E-13		
4	K13777 geranyl-CoA carboxylase alpha subunit	0.02725604	0.050974772	-0.02372	-0.032969162	-0.014468309	1.61E-07	3.81E-07		
**Oxidoreductases/Lyases/Isomerases**										
1	K01825 3-hydroxyacyl-CoA dehydrogenase / enoyl-CoA hydratase / 3-hydroxybutyryl-CoA epimerase / enoyl-CoA isomerase	4.60714239	3.802269179	0.804873	0.714397141	0.895349281	1.38E-66	2.56E-65		
2	K01782 3-hydroxyacyl-CoA dehydrogenase / enoyl-CoA hydratase / 3-hydroxybutyryl-CoA epimerase	2.7600553	2.415050061	0.345005	0.273238123	0.416772349	9.18E-21	4.77E-20		

S/N	Bacterial species	FEN_Res no. Reads	FEN_Res Rel. abundance (%)	FEN_Sus no. reads	FEN_Sus Rel. abundance (%)	Diff. Rel abundance (%)	95% lower CI	95% upper CI	p-values	Corrected p-values

Supplementary Table 3: Significant differences between bacterial taxa detected in FEN_Res and FEN_Sus.

S/N	Bacterial Phylum	FEN_Res Rel. abundance (%)	FEN_Sus Rel. abundance (%)	Diff. Rel abundance (%)	95% lower CI	95% upper CI	p-values	Corrected p-values
1	Firmicutes	0.50065135	0.031911242	0.46874	0.466735802	0.470744405	<1E-301	<1E-301
2	Actinobacteria	0.04860941	0.022241322	0.026368	0.025608474	0.027127703	<1E-301	<1E-301
3	Proteobacteria	99.2648573	99.76198786	-0.49713	-0.499911173	-0.49434999	<1E-301	<1E-301
**4**	**Unclassified Bacteria**	**0.17447182**	**0.172271301**	**0.002201**				

S/N	Bacterial Class	FEN_Res Rel. abundance (%)	FEN_Sus Rel. abundance (%)	Diff. Rel abundance (%)	95% lower CI	95% upper CI	p-values	Corrected p-values
1	Bacilli	0.49259176	0.026219198	0.466373	0.464397032	0.4683481	<1E-301	<1E-301
2	Actinobacteria	0.04780907	0.022163176	0.025646	0.024890711	0.026401082	<1E-301	<1E-301
3	Betaproteobacteria	0.02823082	0.026017532	0.002213	0.001533599	0.002892977	2.06E-10	2.97E-10
4	Flavobacteriia	0	0.010615234	-0.01062	-0.010935832	-0.010294635	<1E-301	<1E-301
5	Alphaproteobacteria	0.04077656	0.056204523	-0.01543	-0.0163485	-0.014507428	3.55E-244	7.70E-244
6	Gammaproteobacteria	99.1740005	99.65739088	-0.48339	-0.486457084	-0.480323735	<1E-301	<1E-301
**7**	**Unclassified Bacteria**	**0.21659132**	**0.201389459**	**0.015202**				

S/N	Bacterial order	FEN_Res Rel. abundance (%)	FEN_SusL Rel. abundance (%)	Diff. Rel abundance (%)	95% lower CI	95% upper CI	p-values	Corrected p-values

S/N	Bacterial species	FEN_Res no. Reads	FEN_Res Rel. abundance (%)	FEN_Sus no. reads	FEN_Sus Rel. abundance (%)	Diff. Rel abundance (%)	95% lower CI	95% upper CI	p-values	Corrected p-values
**1**	Enterobacteriales	91.1723478	87.50930093	3.663047	3.650172	3.675921693	<1E-301	<1E-301		
**2**	Bacillales	0.47581954	0.015609006	0.460211	0.458293145	0.462127925	<1E-301	<1E-301		
**3**	Oceanospirillales	0.01191787	0	0.011918	0.011620031	0.012215711	<1E-301	<1E-301		
**4**	Micrococcales	0.0264538	0.01761307	0.008841	0.008234588	0.009446867	1.05E-174	2.87E-174		
**5**	Pasteurellales	0.0358757	0.029458471	0.006417	0.005674157	0.007160298	2.87E-63	6.16E-63		
**6**	Vibrionales	0.03324795	0.02918622	0.004062	0.003333742	0.004789723	1.59E-27	2.98E-27		
**7**	Uncultured beta proteobacterium CBNPD1 BAC clone 578	0.01601258	0.013410839	0.002602	0.002102711	0.003100771	3.61E-24	6.38E-24		
**8**	Lactobacillales	0.01154386	0.0102724	0.001271	0.000840894	0.001702032	8.82E-09	1.47E-08		
**9**	Flavobacteriales	0	0.010615234	-0.01062	-0.010935832	-0.010294635	<1E-301	<1E-301		
**10**	Rhizobiales	0.0347808	0.0499705	-0.01519	-0.01605137	-0.014328021	3.56E-271	1.07E-270		
**11**	Xanthomonadales	0	0.018570987	-0.01857	-0.018995018	-0.018146956	<1E-301	<1E-301		
**12**	Pseudomonadales	7.78328629	11.94837432	-4.16509	-4.177550389	-4.152625674	<1E-301	<1E-301		
**13**	**Unclassified Bacteria**	**0.38792573**	**0.336528869**	**0.051397**						

S/N	Bacterial family	FEN_Res Rel abundance (%)	FEN_Res Re. abundance (%)	Diff. Rel abundance (%)	95% lower CI	95% upper CI	p-values	Corrected p-values
**1**	Enterobacteriaceae	91.1723458	87.50930093	3.663045	3.650170062	3.675919756	<1E-301	<1E-301
**2**	Bacillaceae	0.4569428	0	0.456943	0.455102676	0.458782917	<1E-301	<1E-301
**3**	Halomonadaceae	0.01129	0	0.01129	0.011000113	0.011579892	<1E-301	<1E-301
**4**	Intrasporangiaceae	0.01079391	0	0.010794	0.010510459	0.011077359	<1E-301	<1E-301
**5**	Pseudomonadaceae	0.03687757	0.027852699	0.009025	0.008287267	0.009762483	2.07E-124	7.24E-124
**6**	Pasteurellaceae	0.0358757	0.029458471	0.006417	0.005674157	0.007160298	2.87E-63	7.41E-63
**7**	Staphylococcaceae	0.01672571	0.012178151	0.004548	0.004055218	0.005039909	1.55E-71	4.48E-71
**8**	Vibrionaceae	0.03323051	0.029145887	0.004085	0.003356991	0.004812259	7.35E-28	1.64E-27

S/N	Bacterial species	FEN_Res no. Reads	FEN_Res Rel. abundance (%)	FEN_Sus no. reads	FEN_Sus Rel. abundance (%)	Diff. Rel abundance (%)	95% lower CI	95% upper CI	p-values	Corrected p-values
9	Uncultured beta proteobacterium CBNPD1 BAC clone 578	0.01601258	0.013410839	0.002602	0.002102711	0.003100771	3.61E-24	7.70E-24		
10	Flavobacteriaceae	0	0.010612713	-0.01061	-0.010933274	-0.010292152	<1E-301	<1E-301		
11	Bradyrhizobiaceae	0.02755838	0.044391893	-0.01683	-0.017630244	-0.016036782	<1E-301	<1E-301		
12	Microbacteriaceae	0	0.01691984	-0.01692	-0.017324585	-0.016515095	<1E-301	<1E-301		
13	Xanthomonadaceae	0	0.018528133	-0.01853	-0.018951674	-0.018104591	<1E-301	<1E-301		
14	Moraxellaceae	7.7454456	11.91954858	-4.1741	-4.186547255	-4.161658712	<1E-301	<1E-301		
**15**	**Unclassified Bacteria**	**0.43690139**	**0.368651862**	**0.06825**						

S/N	Bacterial genera	FEN_Res Rel. abundance (%)	FEN_Sus Rel. abundance (%)	Diff. Rel abundance (%)	95% lower CI	95% upper CI	p-values	Corrected p-values
1	*Klebsiella*	77.5714371	52.42357681	25.14786	25.12859794	25.16712269	<1E-301	<1E-301
2	*Bacillus*	0.45586147	0	0.455861	0.454023516	0.45769942	<1E-301	<1E-301
3	*Escherichia*	2.23880983	1.846133059	0.392677	0.386859521	0.398494024	<1E-301	<1E-301
4	*Pantoea*	0.19576895	0.099865375	0.095904	0.094347753	0.097459406	<1E-301	<1E-301
5	*Raoultella*	0.10322233	0.075690573	0.027532	0.026306998	0.028756509	<1E-301	<1E-301
6	*Salmonella*	0.73754374	0.717666423	0.019877	0.016363096	0.023391532	1.70E-28	3.82E-28
7	*Rahnella*	0.06482159	0.046325373	0.018496	0.017531527	0.019460907	1.54E-301	6.04E-301
8	*Pluralibacter*	0.04399342	0.032400284	0.011593	0.010792503	0.012393759	3.40E-173	1.23E-172
9	*Halomonas*	0.0112745	0	0.011274	0.010984809	0.01156419	<1E-301	<1E-301
10	*Edwardsiella*	0.0348932	0.023756343	0.011137	0.010437103	0.011836612	7.82E-208	2.94E-207
11	*Pseudomonas*	0.03682913	0.027804803	0.009024	0.008287275	0.009761374	1.37E-124	4.44E-124
12	*Providencia*	0.02733553	0.021522884	0.005813	0.005170916	0.006454368	3.26E-69	9.28E-69
13	*Erwinia*	0.03123064	0.025856198	0.005374	0.004679647	0.006069228	3.80E-51	9.65E-51
14	*Haemophilus*	0.01701058	0.01178238	0.005228	0.004737596	0.005718805	2.40E-94	7.53E-94
15	*Photorhabdus*	0.02268271	0.017761799	0.004921	0.004337144	0.005504682	3.75E-60	9.78E-60
16	*Photobacterium*	0.01573934	0.012299151	0.00344	0.00295415	0.00392623	6.02E-43	1.49E-42
17	*Shigella*	0.09749206	0.094304414	0.003188	0.001908037	0.004467254	1.10E-06	1.85E-06

S/N	Bacterial species	FEN_Res no. Reads	FEN_Res Rel. abundance (%)	FEN_Sus no. reads	FEN_Sus Rel. abundance (%)	Diff. Rel abundance (%)	95% lower CI	95% upper CI	p-values	Corrected p-values
**18**	*Staphylococcus*	0.0151948	0.01208488	0.00311	0.002630225	0.003589617	2.53E-36	5.94E-36		
**19**	*Kosakonia*	0.04877025	0.045924561	0.002846	0.001947141	0.003744244	5.99E-10	1.10E-09		
**20**	*Uncultured beta proteobacterium CBNPD1 BAC clone 578*	0.01601258	0.013410839	0.002602	0.002102711	0.003100771	3.61E-24	7.72E-24		
**21**	*Vibrio*	0.01705515	0.016443403	0.000612	7.68E-05	0.001146676	0.025561661	0.0393901		
**22**	*Morganella*	0.01459019	0.015155256	-0.00057	-0.001070374	-5.98E-05	0.028293757	0.042897		
**23**	*Pectobacterium*	0.02095995	0.022332072	-0.00137	-0.001982209	-0.000762035	1.01E-05	1.66E-05		
**24**	*Xenorhabdus*	0.01681098	0.018578549	-0.00177	-0.002319836	-0.001215302	2.97E-10	5.58E-10		
**25**	*Yokenella*	0.02601003	0.027792199	-0.00178	-0.002462342	-0.001102003	2.62E-07	4.56E-07		
**26**	*Yersinia*	0.01403983	0.016271986	-0.00223	-0.002744059	-0.001720246	7.66E-18	1.56E-17		
**27**	*Cedecea*	0.01170858	0.014197339	-0.00249	-0.002962693	-0.002014823	3.05E-25	6.66E-25		
**28**	*Cronobacter*	0.05225841	0.055423065	-0.00316	-0.004126539	-0.00220277	1.03E-10	2.02E-10		
**29**	*Kluyvera*	0.03562765	0.038876308	-0.00325	-0.004049559	-0.002447754	1.47E-15	2.95E-15		
**30**	*Stenotrophomonas*	0	0.015576236	-0.01558	-0.015964581	-0.015187891	<1E-301	<1E-301		
**31**	*Bradyrhizobium*	0.02701384	0.043562539	-0.01655	-0.017337815	-0.015759582	<1E-301	<1E-301		
**32**	*Microbacterium*	0	0.016851778	-0.01685	-0.017255708	-0.016447847	<1E-301	<1E-301		
**33**	*Leclercia*	0.01306315	0.041863497	-0.0288	-0.029509188	-0.028091506	<1E-301	<1E-301		
**34**	*Lelliottia*	0	0.039045204	-0.03905	-0.039659983	-0.038430424	<1E-301	<1E-301		
**35**	*Citrobacter*	0.22675154	0.276066656	-0.04932	-0.051400809	-0.047229418	<1E-301	<1E-301		
**36**	*Serratia*	0.05210144	0.114705527	-0.0626	-0.063827665	-0.061380502	<1E-301	<1E-301		
**37**	*Acinetobacter*	7.7415408	11.91316583	-4.17163	-4.184066422	-4.159183637	<1E-301	<1E-301		
**38**	*Enterobacter*	5.56303539	27.09133651	-21.5283	-21.5434794	-21.51312284	<1E-301	<1E-301		
**39**	**Unclassified Bacteria**	**4.3815093**	**4.674589901**	**-0.29308**						

S/N	Bacterial species	FEN_Res Rel. abundance (%)	FEN_Sus Rel. abundance (%)	Diff. Rel abundance (%)	95% lower CI	95% upper CI	p-values	Corrected p-values
**1**	*Klebsiella pneumoniae*	74.1765919	49.91157543	24.26502	24.24540306	24.28462983	<1E-301	<1E-301
**2**	*Escherichia coli*	2.2137513	1.809107044	0.404644	0.398872188	0.410416318	<1E-301	<1E-301

S/N	Bacterial species	FEN_Res no. Reads	FEN_Res Rel. abundance (%)	FEN_Sus no. reads	FEN_Sus Rel. abundance (%)	Diff. Rel abundance (%)	95% lower CI	95% upper CI	p-values	Corrected p-values
**3**	*Klebsiella sp. MS 92-3*	0.64345651	0.467486215	0.17597	0.172926439	0.179014144	<1E-301	<1E-301		
**4**	*Bacillus anthracis*	0.12361836	0	0.123618	0.122659658	0.124577058	<1E-301	<1E-301		
**5**	*[Enterobacter] aerogenes*	0.30054351	0.204298502	0.096245	0.094194422	0.098295597	<1E-301	<1E-301		
**6**	*Bacillus cereus*	0.0786095	0	0.07861	0.077844827	0.079374176	<1E-301	<1E-301		
**7**	*Klebsiella oxytoca*	0.2672006	0.201253334	0.065947	0.063965165	0.067929374	<1E-301	<1E-301		
**8**	*Klebsiella variicola*	0.23264847	0.169359737	0.063289	0.061454307	0.065123152	<1E-301	<1E-301		
**9**	*Klebsiella quasipneumoniae*	0.08545017	0.047858041	0.037592	0.036543894	0.038640355	<1E-301	<1E-301		
**10**	*Klebsiella sp. 1_1_55*	0.12762393	0.091508809	0.036115	0.034760797	0.037469436	<1E-301	<1E-301		
**11**	*Acinetobacter johnsonii*	0.05679689	0.024116823	0.03268	0.031870098	0.033490039	<1E-301	<1E-301		
**12**	*Salmonella enterica*	0.73317385	0.709826628	0.023347	0.019848245	0.0268462	5.66E-39	1.03E-38		
**13**	*Klebsiella michiganensis*	0.09768391	0.077387095	0.020297	0.019082201	0.021511426	4.38E-231	1.04E-230		
**14**	*Raoultella ornithinolytica*	0.07753592	0.058117837	0.019418	0.018350747	0.020485427	1.62E-272	3.93E-272		
**15**	*Rahnella aquatilis*	0.06398637	0.045473331	0.018513	0.01755587	0.019470209	9.65E-307	2.36E-306		
**16**	*Klebsiella sp. OBRC7*	0.03260071	0.015462798	0.017138	0.016511552	0.017764264	<1E-301	<1E-301		
**17**	*Citrobacter sp. BIDMC108*	0.0161056	0	0.016106	0.015759369	0.016451826	<1E-301	<1E-301		
**18**	*Pantoea sp. FF5*	0.01591762	0	0.015918	0.015573422	0.016261827	<1E-301	<1E-301		
**19**	*Klebsiella sp. 4_1_44FAA*	0.04023783	0.027872866	0.012365	0.011610476	0.013119457	1.66E-220	3.85E-220		
**20**	*Bacillus thuringiensis*	0.01177447	0	0.011774	0.011478426	0.012070512	<1E-301	<1E-301		
**21**	*Citrobacter sp. MGH99*	0.01158456	0	0.011585	0.011290912	0.011878204	<1E-301	<1E-301		
**22**	*Pluralibacter gergoviae*	0.04397791	0.032400284	0.011578	0.010777072	0.012378184	8.95E-173	1.97E-172		
**23**	*Halomonas titanicae*	0.01118148	0	0.011181	0.010892989	0.011469975	<1E-301	<1E-301		
**24**	*Edwardsiella ictaluri*	0.02704097	0.016657674	0.010383	0.009781196	0.010985398	1.98E-242	4.75E-242		
**25**	*Klebsiella sp. 10982*	0.02454694	0.015263652	0.009283	0.008708417	0.009858155	5.93E-213	1.36E-212		
**26**	*Citrobacter sp. BIDMC107*	0.02624063	0.019357487	0.006883	0.006264513	0.007501777	4.67E-103	9.60E-103		
**27**	*Kosakonia radicincitans*	0.04559022	0.039559454	0.006031	0.005180963	0.006880562	1.76E-43	3.26E-43		
**28**	*Photorhabdus luminescens*	0.02168665	0.016516507	0.00517	0.004603292	0.005736993	5.32E-70	1.03E-69		
**29**	*Shigella sonnei*	0.04620258	0.041490413	0.004712	0.003848805	0.005575532	1.81E-26	3.16E-26		
**30**	*Raoultella planticola*	0.01490412	0.011137046	0.003767	0.003299348	0.004234802	6.25E-55	1.17E-54		
**31**	*Erwinia sp. 9145*	0.01633814	0.013597381	0.002741	0.002237511	0.00324401	3.19E-26	5.52E-26		

S/N	Bacterial species	FEN_Res no. Reads	FEN_Res Rel. abundance (%)	FEN_Sus no. reads	FEN_Sus Rel. abundance (%)	Diff. Rel abundance (%)	95% lower CI	95% upper CI	p-values	Corrected p-values
**32**	*uncultured beta proteobacterium CBNPD1 BAC clone 578*	0.01601258	0.013410839	0.002602	0.002102711	0.003100771	3.61E-24	6.06E-24		
**33**	*Photobacterium sp. SKA34*	0.01378985	0.011290817	0.002499	0.002038635	0.002959428	5.24E-26	8.99E-26		
**34**	*Klebsiella sp. KTE92*	0.01257287	0.010300129	0.002273	0.001833062	0.002712418	9.85E-24	1.64E-23		
**35**	*Klebsiella sp. AS10*	0.01407859	0.012140338	0.001938	0.001466724	0.00240978	1.19E-15	1.93E-15		
**36**	*Providencia stuartii*	0.0144836	0.013158755	0.001325	0.000839862	0.001809836	9.93E-08	1.51E-07		
**37**	*Morganella morganii*	0.01457662	0.015147694	-0.00057	-0.001076205	-6.59E-05	0.026420352	0.0362241		
**38**	*Shigella dysenteriae*	0.01367551	0.014855277	-0.00118	-0.001675365	-0.00068416	2.86E-06	4.24E-06		
**39**	*Citrobacter youngae*	0.02076035	0.02196403	-0.0012	-0.001809616	-0.000597744	9.57E-05	0.0001381		
**40**	*Xenorhabdus cabanillasii*	0.0138693	0.015450194	-0.00158	-0.002083708	-0.001078077	6.07E-10	9.58E-10		
**41**	*Yokenella regensburgei*	0.02601003	0.027792199	-0.00178	-0.002462342	-0.001102003	2.62E-07	3.94E-07		
**42**	*Pectobacterium carotovorum*	0.0115458	0.014053652	-0.00251	-0.002979032	-0.00203667	6.40E-26	1.09E-25		
**43**	*Kluyvera cryocrescens*	0.0230761	0.025634365	-0.00256	-0.003206275	-0.001910257	7.52E-15	1.21E-14		
**44**	*Cronobacter sakazakii*	0.04617739	0.048858812	-0.00268	-0.003585026	-0.00177782	5.56E-09	8.73E-09		
**45**	*Citrobacter sp. 30_2*	0.01915774	0.024366385	-0.00521	-0.005823862	-0.004593438	3.08E-63	5.84E-63		
**46**	*Acinetobacter oleivorans*	0.01022418	0.017091257	-0.00687	-0.00735859	-0.006375571	4.12E-174	9.16E-174		
**47**	*Acinetobacter sp. WC-141*	0.01207871	0.020015425	-0.00794	-0.008469337	-0.007404086	1.45E-197	3.26E-197		
**48**	*Citrobacter freundii*	0.03024039	0.039503996	-0.00926	-0.010042995	-0.008484224	3.84E-123	8.12E-123		
**49**	*Enterobacter sp. MGH 24*	0	0.010184171	-0.01018	-0.010498194	-0.009870148	<1E-301	<1E-301		
**50**	*Shigella boydii*	0	0.010239629	-0.01024	-0.010554506	-0.009924753	<1E-301	<1E-301		
**51**	*Enterobacter sp. MGH 25*	0	0.0102724	-0.01027	-0.01058778	-0.00995702	<1E-301	<1E-301		
**52**	*Enterobacter sp. GN03164*	0	0.010345504	-0.01035	-0.010662004	-0.010029004	<1E-301	<1E-301		
**53**	*Acinetobacter ursingii*	0	0.010549692	-0.01055	-0.0108693	-0.010230084	<1E-301	<1E-301		
**54**	*Acinetobacter sp. NIPH 542*	0	0.010612713	-0.01061	-0.010933274	-0.010292152	<1E-301	<1E-301		
**55**	*Enterobacter sp. MGH 14*	0	0.01087488	-0.01087	-0.011199375	-0.010550384	<1E-301	<1E-301		
**56**	*Acinetobacter haemolyticus*	0	0.010978234	-0.01098	-0.011304268	-0.0106522	<1E-301	<1E-301		
**57**	*Acinetobacter sp. ATCC 27244*	0	0.011066463	-0.01107	-0.011393804	-0.010739122	<1E-301	<1E-301		
**58**	*Enterobacter sp. SENG-6*	0	0.011142088	-0.01114	-0.011470546	-0.01081363	<1E-301	<1E-301		
**59**	*Acinetobacter junii*	0	0.011777338	-0.01178	-0.012115028	-0.011439648	<1E-301	<1E-301		
**60**	*Stenotrophomonas maltophilia*	0	0.012248734	-0.01225	-0.012593115	-0.011904353	<1E-301	<1E-301		

S/N	Bacterial species	FEN_Res no. Reads	FEN_Res Rel. abundance (%)	FEN_Sus no. reads	FEN_Sus Rel. abundance (%)	Diff. Rel abundance (%)	95% lower CI	95% upper CI	p-values	Corrected p-values
**61**	*Acinetobacter seifertii*	0	0.013738547	-0.01374	-0.014103268	-0.013373826	<1E-301	<1E-301		
**62**	*Enterobacter sp. 50858885*	0	0.013859547	-0.01386	-0.014225871	-0.013493224	<1E-301	<1E-301		
**63**	*Acinetobacter sp. WC-136*	0	0.014194818	-0.01419	-0.014565546	-0.013824091	<1E-301	<1E-301		
**64**	*Acinetobacter sp. LMB-5*	0	0.014454464	-0.01445	-0.014828566	-0.014080362	<1E-301	<1E-301		
**65**	*Bradyrhizobium sp. DFCI-1*	0	0.01495359	-0.01495	-0.015334095	-0.014573084	<1E-301	<1E-301		
**66**	*Enterobacter sp. MGH 23*	0	0.016279548	-0.01628	-0.016676563	-0.015882534	<1E-301	<1E-301		
**67**	*Microbacterium sp. SUBG005*	0	0.016801361	-0.0168	-0.017204687	-0.016398035	<1E-301	<1E-301		
**68**	*Enterobacter sp. 35699*	0	0.017287882	-0.01729	-0.017697005	-0.016878759	<1E-301	<1E-301		
**69**	*Enterobacter sp. T1-1*	0	0.019725529	-0.01973	-0.020162539	-0.019288519	<1E-301	<1E-301		
**70**	*Enterobacter cloacae complex sp. GN06232*	0	0.020020467	-0.02002	-0.020460731	-0.019580202	<1E-301	<1E-301		
**71**	*Enterobacter sp. FY-07*	0.09164358	0.112308213	-0.02066	-0.021994265	-0.019334997	1.03E-207	2.34E-207		
**72**	*Enterobacter sp. BIDMC92*	0	0.020701092	-0.0207	-0.021148776	-0.020253408	<1E-301	<1E-301		
**73**	*Enterobacter sp. 638*	0.01357281	0.034507702	-0.02093	-0.021594494	-0.020275294	<1E-301	<1E-301		
**74**	*Citrobacter koseri*	0.04706687	0.072365593	-0.0253	-0.026323647	-0.024273799	<1E-301	<1E-301		
**75**	*Acinetobacter sp. P8-3-8*	0.01995032	0.047061457	-0.02711	-0.027888309	-0.026333961	<1E-301	<1E-301		
**76**	*Leclercia adecarboxylata*	0.01294106	0.040782059	-0.02784	-0.028541768	-0.027140222	<1E-301	<1E-301		
**77**	*Enterobacter sp. DC3*	0	0.028117387	-0.02812	-0.028639118	-0.027595656	<1E-301	<1E-301		
**78**	*Enterobacter sp. MT20*	0	0.029506366	-0.02951	-0.030040825	-0.028971908	<1E-301	<1E-301		
**79**	*Enterobacter xiangfangensis*	0	0.032208701	-0.03221	-0.03276709	-0.031650312	<1E-301	<1E-301		
**80**	*Enterobacter ludwigii*	0	0.034479973	-0.03448	-0.035057708	-0.033902237	<1E-301	<1E-301		
**81**	*Acinetobacter soli*	0.01168726	0.046607707	-0.03492	-0.035654006	-0.034186878	<1E-301	<1E-301		
**82**	*Acinetobacter nosocomialis*	0.01042959	0.046065727	-0.03564	-0.036359679	-0.034912595	<1E-301	<1E-301		
**83**	*Acinetobacter calcoaceticus*	0.05292891	0.091339913	-0.03841	-0.039541269	-0.037280734	<1E-301	<1E-301		
**84**	*Lelliottia amnigena*	0	0.039045204	-0.03905	-0.039659983	-0.038430424	<1E-301	<1E-301		
**85**	*Serratia marcescens*	0.0299342	0.07716274	-0.04723	-0.048213125	-0.046243949	<1E-301	<1E-301		
**86**	*Enterobacter sp. GN02548*	0	0.055720523	-0.05572	-0.056454879	-0.054986167	<1E-301	<1E-301		
**87**	*Enterobacter sp. UCD-UG_FMILLET*	0	0.056454086	-0.05645	-0.057193258	-0.055714914	<1E-301	<1E-301		
**88**	*Enterobacter mori*	0	0.057333857	-0.05733	-0.058078763	-0.056588951	<1E-301	<1E-301		
**89**	*Enterobacter sp. DC1*	0	0.061173088	-0.06117	-0.061942515	-0.060403661	<1E-301	<1E-301		

S/N	Bacterial species	FEN_Res no. Reads	FEN_Res Rel. abundance (%)	FEN_Sus no. reads	FEN_Sus Rel. abundance (%)	Diff. Rel abundance (%)	95% lower CI	95% upper CI	p-values	Corrected p-values
**90**	*Enterobacter sp. GN02283*	0	0.06555934	-0.06556	-0.066355857	-0.064762823	<1E-301	<1E-301		
**91**	*Enterobacter cloacae complex sp. GN04787*	0.01449329	0.114773589	-0.10028	-0.10138394	-0.099176651	<1E-301	<1E-301		
**92**	*Enterobacter sp. EGD-HP1*	0.0243299	0.12815922	-0.10383	-0.105021185	-0.10263746	<1E-301	<1E-301		
**93**	*Enterobacter kobei*	0.01764039	0.122845301	-0.1052	-0.106353578	-0.10405625	<1E-301	<1E-301		
**94**	*Enterobacter cancerogenus*	0.03225577	0.163022359	-0.13077	-0.13211423	-0.129418957	<1E-301	<1E-301		
**95**	*Enterobacter sp. 50588862*	0.0208243	0.181870638	-0.16105	-0.162429438	-0.15966324	<1E-301	<1E-301		
**96**	*Enterobacter sp. BIDMC 29*	0.02562245	0.187199682	-0.16158	-0.16299147	-0.160162987	<1E-301	<1E-301		
**97**	*Enterobacter cloacae complex 'Hoffmann cluster IV'*	0.07477834	0.288375891	-0.2136	-0.215425318	-0.21176978	<1E-301	<1E-301		
**98**	*Enterobacter sp. 35669*	0.03824183	0.287929703	-0.24969	-0.251438518	-0.247937225	<1E-301	<1E-301		
**99**	*Enterobacter hormaechei*	0.28776329	0.668401757	-0.38064	-0.383565127	-0.377711807	<1E-301	<1E-301		
**100**	*Acinetobacter baumannii*	2.1325063	3.144309828	-1.0118	-1.018513777	-1.00509328	<1E-301	<1E-301		
**101**	*Enterobacter asburiae*	0.28542816	1.381336574	-1.09591	-1.099821259	-1.091995564	<1E-301	<1E-301		
**102**	*Acinetobacter pittii*	3.2170403	5.16661896	-1.94958	-1.957982522	-1.941174799	<1E-301	<1E-301		
**103**	*Enterobacter cloacae*	2.75678182	16.02785572	-13.2711	-13.28333312	-13.25881469	<1E-301	<1E-301		
**104**	**Unclassified Bacteria**	**10.760322**	**16.36087811**	**-5.60056**						
